# Short- and long-term outcomes in isolated vs. hybrid thoracoscopic ablation in patients with atrial fibrillation: a systematic review and reconstructed individual patient data meta-analysis

**DOI:** 10.1093/europace/euae232

**Published:** 2024-09-10

**Authors:** Luca Aerts, Michal J Kawczynski, Elham Bidar, Justin G L Luermans, Sevasti-Maria Chaldoupi, Mark La Meir, Mariusz Kowalewski, Jos G Maessen, Samuel Heuts, Bart Maesen

**Affiliations:** Department of Cardiothoracic Surgery, Maastricht University Medical Centre, Postbus 5800, 6202 AZ Maastricht, The Netherlands; Cardiovascular Research Institute Maastricht (CARIM), Maastricht University, PO Box 616, 6200 MD Maastricht, The Netherlands; Department of Cardiothoracic Surgery, Maastricht University Medical Centre, Postbus 5800, 6202 AZ Maastricht, The Netherlands; Cardiovascular Research Institute Maastricht (CARIM), Maastricht University, PO Box 616, 6200 MD Maastricht, The Netherlands; Department of Cardiothoracic Surgery, Maastricht University Medical Centre, Postbus 5800, 6202 AZ Maastricht, The Netherlands; Cardiovascular Research Institute Maastricht (CARIM), Maastricht University, PO Box 616, 6200 MD Maastricht, The Netherlands; Cardiovascular Research Institute Maastricht (CARIM), Maastricht University, PO Box 616, 6200 MD Maastricht, The Netherlands; Department of Cardiology, Maastricht University Medical Centre, Postbus 5800, 6202 AZ Maastricht, The Netherlands; Cardiovascular Research Institute Maastricht (CARIM), Maastricht University, PO Box 616, 6200 MD Maastricht, The Netherlands; Department of Cardiology, Maastricht University Medical Centre, Postbus 5800, 6202 AZ Maastricht, The Netherlands; Department of Cardiac Surgery, University Hospital Brussels, Brussels, Belgium; Cardiovascular Research Institute Maastricht (CARIM), Maastricht University, PO Box 616, 6200 MD Maastricht, The Netherlands; Clinical Department of Cardiac Surgery and Transplantology, National Medical Institute of the Ministry of Interior and Administration, Centre of Postgraduate Medical Education, Warsaw, Poland; Department of Cardiothoracic Surgery, Maastricht University Medical Centre, Postbus 5800, 6202 AZ Maastricht, The Netherlands; Cardiovascular Research Institute Maastricht (CARIM), Maastricht University, PO Box 616, 6200 MD Maastricht, The Netherlands; Department of Cardiothoracic Surgery, Maastricht University Medical Centre, Postbus 5800, 6202 AZ Maastricht, The Netherlands; Cardiovascular Research Institute Maastricht (CARIM), Maastricht University, PO Box 616, 6200 MD Maastricht, The Netherlands; Department of Cardiothoracic Surgery, Maastricht University Medical Centre, Postbus 5800, 6202 AZ Maastricht, The Netherlands; Cardiovascular Research Institute Maastricht (CARIM), Maastricht University, PO Box 616, 6200 MD Maastricht, The Netherlands

**Keywords:** Thoracoscopic ablation, Hybrid ablation, Hybrid thoracoscopic ablation, Atrial fibrillation, Ablation

## Abstract

**Aims:**

Both isolated thoracoscopic and hybrid thoracoscopic atrial fibrillation (AF) ablation techniques have demonstrated favourable outcomes in the management of patients with (long-standing) persistent AF, as compared with catheter ablation. However, it is currently unknown whether there is a difference in short- and long-term outcomes when comparing these two minimally invasive surgical AF ablation procedures. Therefore, a systematic review and meta-analysis were performed to investigate these two techniques, with a specific emphasis on long-term freedom from atrial tachyarrhythmias (ATAs).

**Methods and results:**

A systematic search through PubMed, EMBASE, and the Cochrane Library databases was performed. All studies reporting on short-term outcomes were included in the meta-analysis. A pooled analysis of long-term freedom from ATA was performed based on Kaplan–Meier (KM) curve-derived individual patient data. Reconstructed individual time-to-event data were analysed in a multivariable Cox frailty model with adjustments for age, sex, type of AF, duration of AF history, and study variable (frailty term in the frailty Cox model). In total, 53 studies were included in the meta-analysis, encompassing 4950 patients. There were no differences in major short-term outcomes (mortality or stroke) between isolated thoracoscopic and hybrid thoracoscopic ablation. A total of 18 studies reported KM curves for long-term freedom from ATA, comprising 2038 patients. Adjusted analysis revealed that hybrid ablation was significantly associated with greater freedom from ATA [adjusted hazard ratio (aHR) = 0.59, 95% confidence interval (CI): 0.43–0.83, *P* < 0.001] compared with isolated thoracoscopic ablation. Additionally, older age (aHR = 1.07, 95% CI: 1.03–1.12, *P* = 0.002) and a higher percentage of male patients (aHR = 1.02, 95% CI: 1.01–1.03, *P* < 0.001) were significantly associated with lower long-term freedom from ATA recurrence.

**Conclusion:**

Hybrid thoracoscopic AF ablation is associated with a greater long-term freedom from ATA when compared with isolated thoracoscopic ablation, without differences in complications.

## Graphical Abstract

**Graphical Abstract:**
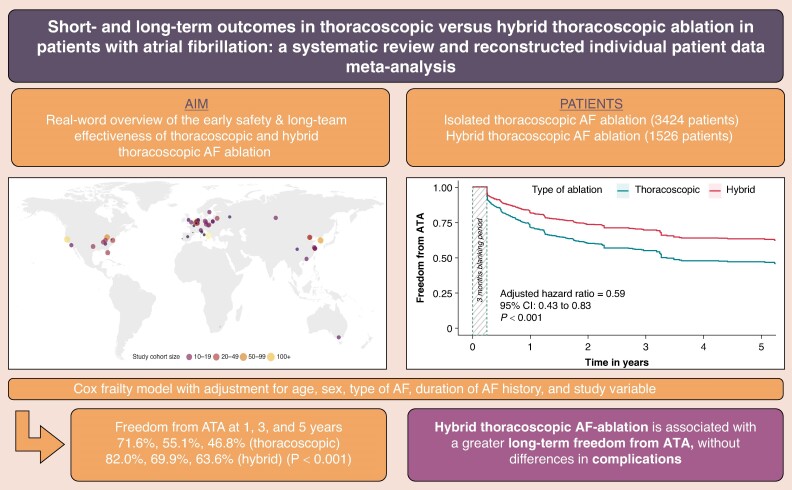

## Introduction

The management of atrial fibrillation (AF), particularly in (long-standing) persistent AF, poses a clinical challenge for both patients and physicians. Catheter ablation has proven its efficacy in patients with paroxysmal AF (pAF) with a success rate of 80%.^1^ However, its effectiveness decreases when applied to persistent forms of AF.^2^ Although the results of the isolated thoracoscopic AF ablation are favourable compared with catheter ablation,^3^ a limitation lies in the surgeon’s lack of visibility into the underlying electrophysiological properties of the atria. Additionally, not all lesions can be exclusively addressed epicardially, such as the mitral or cavo-tricuspid isthmus line. Consequently, *hybrid* thoracoscopic ablation was developed, combining thoracoscopic epicardial ablation with transvenous endocardial catheter mapping and ablation.^4,5^

Despite the theoretical advantages and the promising success rates of the 1-year follow-up of freedom from atrial tachyarrhythmias (ATAs) of the hybrid thoracoscopic approach (71%),^6^ evidence is lacking when comparing this procedure to a solely epicardial thoracoscopic approach. One meta-analysis suggested that there was no difference in outcome between thoracoscopic and hybrid thoracoscopic ablation^7^; however, the long-term efficacy and safety of these procedures using Kaplan–Meier (KM) curve-derived individual patient data (IPD) have never been compared. Therefore, this meta-analysis aims to investigate the early safety and long-term effectiveness of both thoracoscopic and hybrid thoracoscopic ablations.

## Methods

### Design

This study was designed as a systemic review and meta-analysis to investigate the short- and long-term outcomes in patients undergoing standalone thoracoscopic and hybrid thoracoscopic AF ablation. The study was registered in PROSPERO^8^ with registration date 27 November 2022 (CRD42022376286) and followed the 2020 Preferred Reporting Items for Systematic Reviews and Meta-Analysis (PRISMA 2020) guidelines.^9^

### Eligibility and exclusion criteria

Studies meeting the specific criteria for this meta-analysis included a publication date between 1 January 2012 and 6 September 2023, consecutive patients undergoing thoracoscopic AF ablation (both thoracoscopic and hybrid thoracoscopic), and reporting on the early outcomes, preferably along with long-term freedom from ATAs. All studies were checked for a possible overlap of study populations. In case of overlap, the study with the largest population was included in the data synthesis. Both single- and multicentre studies were deemed eligible for inclusion. To minimize the risk of publication bias, abstracts, and poster presentations were also included when describing the aforementioned inclusion criteria. Studies reporting on the convergent procedure, the open Cox maze procedure, thoracoscopic ablation procedures using the Cobra Fusion device, and thoracoscopic ablation concomitant with another cardiothoracic procedure were excluded from the analyses.

### Information sources

A systematic search through PubMed, EMBASE, and the Cochrane Library databases was performed using the following search terms: ‘thoracoscopic ablation’, ‘hybrid ablation’, ‘atrial fibrillation’, and various alternative spellings. Please see Supplementary material online, *Material S1* for the comprehensive search strategy.

### Selection process

The selection process was carried out independently by two authors (L.A. and B.M.). Initial screening of studies for eligibility was conducted based on the title and abstract. For the study selection process, a web-based tool (Rayyan; http://rayyan.qcri.org) was used. Subsequently, full texts meeting the aforementioned criteria were further assessed for final eligibility. Any disagreements were resolved through discussion.

### Data collection process and items

Data collection was conducted manually and independently by L.A. and M.J.K., using a pre-defined worksheet (see Supplementary material online, *Table S1*). Any disagreements were resolved by consulting the senior author (B.M.).

### Outcomes and effect measures

The primary outcome was short-term adverse events, while the secondary outcome concerned long-term freedom from ATA. The difference in long-term freedom from ATA for isolated thoracoscopic and hybrid ablation was presented in reconstructed KM curves along with (adjusted) hazard ratios (HRs) and corresponding 95% confidence intervals (CIs; please see *Data synthesis*). Differences in baseline characteristics and short-term outcomes were presented in tables.

### Risk of bias assessment

The Newcastle–Ottawa scale was employed for an objective evaluation of the study quality. The assessed items cover Selection (representativeness of the exposed cohort, selection of the non-exposed cohort, ascertainment of exposure, and outcome of interest not present at the start of the study), Comparability (comparability of cohorts on the basis of design or analysis), and Outcome (assessment of outcome, long enough follow-up, and adequacy of follow-up). The scale was adaptable for single-arm studies,^10^ involving modification in the selection process (i.e. Question 2 in Selection—selection of the non-exposed cohort). Two authors (L.A. and M.J.K.) conducted this quality assessment. Any disagreements were resolved by consulting the senior author (B.M.).

### Data synthesis

Data from studies presenting medians and inter-quartile ranges were converted to mean and standard deviation using Wan’s method.^11^ Complete case analysis was performed, and no imputation techniques were applied in case of missing values, because of presumable non-random missingness of data.

Study and patient characteristics were presented in tables with weighted pooled mean values and corresponding 95% CI. Patient characteristics and short-term outcomes were pooled in a meta-analysis of single means (continuous variables) or proportions (categorical variables) using inverse variance weighting in a random effects model. Comparisons between patient characteristics for studies reporting on hybrid thoracoscopic vs. isolated thoracoscopic ablation were assessed with a meta-analysis of continuous or categorical variables using inverse variance weighting in a random effects model. Between-study variance (*τ*^2^) was assessed with the restricted maximum likelihood estimator. Additionally, statistical heterogeneity was assessed using the *I*^2^ metric, with *I*^2^ > 75% implying high between-study heterogeneity, for which a *P*-value <0.05 indicated the presence of statistically significant between-study heterogeneity.

For assessment of the long-term freedom from ATA, a meta-analysis of reconstructed KM-derived IPD was performed, as proposed by Liu *et al*.^12^ All studies included in the this analysis reported KM curves for long-term freedom from ATA, from which IPD could be derived and presented in an unadjusted cumulative KM curve. Adjusted analyses were performed in a multivariable Cox frailty model, with adjustment for the study parameter, representing additional heterogeneity not accounted for by the variables included in the model.^13^ Clinical variables included in the model were chosen based on previous literature reports and on results from the unadjusted baseline group comparisons between patients undergoing hybrid thoracoscopic vs. isolated thoracoscopic ablation. The reasons for the inclusion of certain variables in the adjusted analyses were substantiated and presented along with the models in the Supplementary material. The multivariable frailty model included adjustments for age, sex, AF history, and a frailty term (study variable). The proportional hazard assumption was assessed visually and tested statically by Schoenfeld residuals.

All statistical analyses were performed with R version 4.2.2. (R foundation, Vienna, Austria) using ‘metafor’, ‘meta’, ‘survival’, ‘survminer’, ‘maps’, and ‘ggplot2’ packages. A significance threshold of *P* < 0.05 was deemed as statistically significant.

### Publication bias assessment and adjustment

Publication bias was assessed for patient characteristics and short-term outcomes statistically using Egger’s test. No publication bias corrections were performed, but the presence of significant publication bias was reported and explored in the results section.

## Results

### Study selection

**Figure 1:**
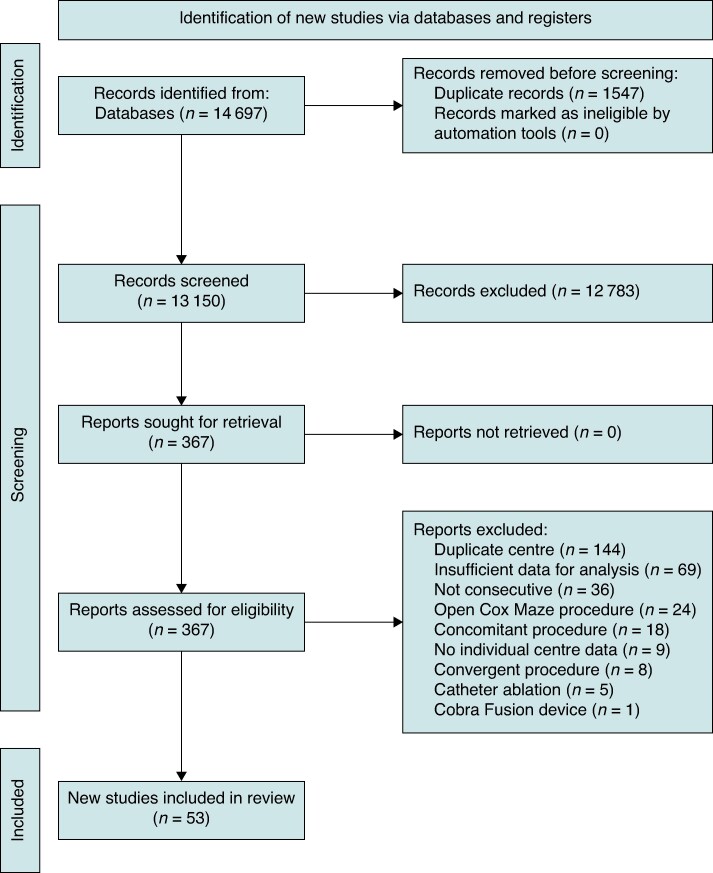
Preferred Reporting Items for Systematic Reviews and Meta-Analyses 2020 flowchart for study inclusion.

The systematic search initially identified a total of 14697 potentially eligible studies. After removing duplicates, 13 150 unique studies remained. Subsequently, based on an assessment of titles and abstracts, 12 783 studies were excluded, leaving 367 studies for screening of full texts. All full-text reports were retrieved, and based on full-text assessment, 53 studies were included for the final analysis (see the PRISMA flowchart in *Figure 1* for detailed information regarding the exclusion process).

### Study characteristics

**Figure 2:**
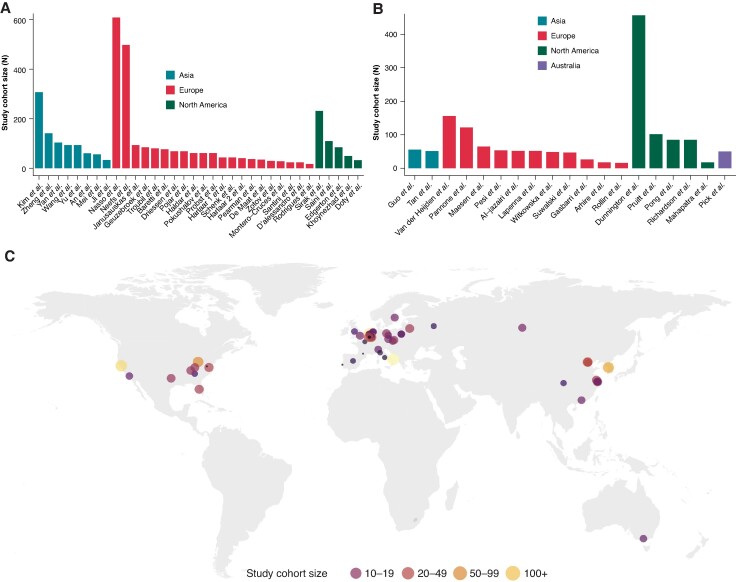
Study sample size and geographical distribution. (A) Sample size per study reporting on thoracoscopic ablation. (B) Sample size per study reporting on hybrid thoracoscopic ablation. (C) Geographical distribution.

A total of 53 studies were included, originating from 48 individual centres across 4 continents and 24 countries, involving 4950 patients undergoing isolated thoracoscopic ablation (34 studies, *n* = 3424) and hybrid thoracoscopic ablation (19 studies, *n* = 1526). Study characteristics regarding the type of study, follow-up duration, and post-operative monitoring strategies, along with ATA recurrence definitions, are presented in Supplementary material online, *Table S2*. The majority of studies had a retrospective design (retrospective: 32 studies; prospective: 21 studies). There were no randomized controlled trials (RCTs). Sample sizes ranged from 15 to 609 patients per study, reporting on thoracoscopic ablation (*Figure 2A*) and from 13 to 363 patients per study, reporting on hybrid thoracoscopic ablation (*Figure 2B*). *Figure 2C* displays the geographical distribution of the studies. The majority of studies originated from Europe (*n* = 32), followed by Asia (*n* = 10) and North America (*n* = 10). One study originated from Australia.

### Study quality assessment

Overall study quality ranged from low to high risk of bias, with the majority of studies having a low risk of bias (low risk of bias: *n* = 38 studies; moderate to high risk of bias: *n* = 13; and unable to assess risk of bias due to insufficient data: *n* = 2). The study quality assessment is presented in Supplementary material online, *Table S3*, with a description of domain-specific grading per study. Because of the uniform reporting on the primary outcome, all studies were included for final analysis.

### Overall patient and procedural characteristics

Pooled patient and procedural characteristics of all studies included in the meta-analysis (*n* = 53) for patients undergoing thoracoscopic or hybrid thoracoscopic ablation are presented in *Table 1*. The mean age of the study population was 59.9 years old (95% CI: 58.8–61.0 years old), with the minority of patients being female (26.5%, 95% CI: 23.6–29.5%) and a minority of patients with a history of PAF (19.5%, 95% CI: 11.9–30.3%). The average AF duration was 6.2 years (95% CI: 5.4–7.0 years). Also, one-third of patients underwent catheter ablation prior to surgical AF ablation (32.0%, 95% CI: 24.1–41.1%).

**Table 1:** Patient and procedural characteristics

Variables	Overall (*n* = 4 950)	Thoracoscopic (*n* = 3 424)	Hybrid (*n* = 1 526)	*P*-value^a^
Age, in years	59.9 (58.8–61.0)	59.6 (58.0–61.1)	60.5 (58.8–63.2)	0.545
Female subjects, in %	26.5 (23.6–29.5)	26.1 (22.7–29.9)	27.1 (22.2–32.6)	0.151
BMI, in kg/m^2^	29.7 (27.9–31.4)	30.2 (27.2–33.2)	29.1 (27.4–30.9)	0.561
PAF history, in %	19.5 (11.9–30.3)	27.4 (16.5–41.9)	8.2 (3.0–20.2)	**<0**.**001**
AF duration, in years	6.2 (5.4–7.0)	5.9 (4.8–7.0)	6.7 (5.8–7.7)	0.122
CHA₂DS₂-VASc score	2 (2–2)	2 (1–2)	2 (2–2)	0.131
Hypertension, in %	55.3 (48.2–62.2)	54.7 (45.5–63.5)	58.0 (47.9–67.5)	0.059
Diabetes mellitus, in %	9.8 (7.8–12.2)	8.8 (6.4–12.1)	12.2 (9.5–15.5)	**0**.**001**
Stroke history, in %	10.3 (8.5–12.4)	10.7 (8.7–13.1)	9.4 (6.4–13.7)	0.747
Prior PVI, in %	32.0 (24.1–41.1)	29.6 (19.6–42.0)	35.5 (25.5–46.9)	0.055
LVEF, in %	56.9 (55.4–58.5)	58.0 (55.8–60.2)	55.8 (53.8–57.8)	0.158
LAD, in mm	45.7 (44.2–47.2)	44.7 (43.2–46.3)	47.5 (44.6–50.4)	0.144
Procedure time, in min	200.4 (158.4–242.4)	183.7 (147.1–220.4)	226.9 (133.1–320.8)	0.339
Conversion to sternotomy, in %	2.1 (1.4–3.1)	2.4 (1.6–3.8)	1.6 (0.1–3.3)	0.200

AF, atrial fibrillation; BMI, body mass index; LAD, left atrial diameter; LVEF, left ventricular ejection fraction; PAF, paroxysmal atrial fibrillation; PVI, pulmonary vein isolation.

^a^Obtained from weighted group comparison analysis.

Bold values are significant values.

The majority of demographic characteristics of patients included in the studies of thoracoscopic ablation did not differ significantly from those in the studies on hybrid thoracoscopic ablation (*Table 1*). However, there was a significant difference between thoracoscopic and hybrid thoracoscopic patients in PAF history (27.4% vs. 8.2%, respectively, *P* < 0.001) and diabetes mellitus (8.8% vs. 12.2%, respectively, *P* = 0.001) (*Table 1*). There was no significant difference in procedural duration between both groups (*Table 1*).

In the studies reporting on thoracoscopic ablation, a minority of patients (*n* = 1026) received pulmonary vein isolation (PVI) with box lesion only (20/34 studies), while a majority of patients (*n* = 2398) received additional epicardial lesions (14/34 studies). In the hybrid thoracoscopic patient group, PVI with box lesion and endocardial validation was performed in 215 patients (5/19 studies), additional endocardial lesions were performed in 427 patients (6/19 studies), additional epicardial lesions were performed in 652 patients (4/19 studies), and additional epicardial and endocardial lesions were performed in 232 patients (4/19 studies). For studies specifying left atrial appendage (LAA) management, the LAA was excluded in 95.8% of patients in the thoracoscopic group and 96.4% of patients in the hybrid thoracoscopic group. Overall, the LAA was excluded in 46.3% with a stapler, in 31.9% with a clipping device, and in 17.8% with another technique. No LAA management was performed in 4% (see Supplementary material online, *Table S4*).

### Overall short-term outcomes

Pooled short-term outcomes of all studies included in the meta-analysis (*n* = 53) for patients undergoing isolated thoracoscopic or hybrid thoracoscopic ablation are presented in *Table 2*. The overall pooled early mortality (0.1%, 95% CI: 0.0–0.1%) and early stroke rates (0.0%, 95% CI: 0.0–0.1%) were very low. There were no significant differences in short-term outcomes between isolated thoracoscopic and hybrid thoracoscopic ablation, except for early pleural effusion, which occurred more frequently in hybrid thoracoscopic ablation (4.4%, 95% CI: 1.6–11.4%) compared with thoracoscopic ablation (1.9%, 95% CI: 0.1–4.9%) (*P* = 0.019; *Table 2*).

**Table 2:** Short-term outcomes for hybrid vs. thoracoscopic ablation

Variables	Overall (*n* = 4 950)	Thoracoscopic (*n* = 3 424)	Hybrid (*n* = 1 526)	*P*-value^a^
Early mortality, in %	0.1 (0.0–0.1)	0.0 (0.0–0.0)	1.0 (0.6–1.8)	0.062
Early stroke, in %	0.0 (0.0–0.1)	1.9 (1.2–3.1)	0.1 (0.0–0.2)	0.074
Early reoperations, in %	2.4 (1.7–3.3)	2.3 (1.5–3.4)	1.9 (0.1–3.5)	0.592
Cardiac tamponade, in %	2.9 (1.7–4.7)	4.2 (2.2–7.9)	2.0 (1.0–3.8)	0.302
Any bleeding, in %	3.5 (2.2–5.5)	2.9 (1.5–5.5)	4.1 (2.1–7.9)	0.823
Pneumonia, in %	2.4 (1.3–4.4)	2.2 (0.1–5.3)	2.8 (1.2–6.7)	0.370
Pleural effusion, in %	2.9 (1.5–5.7)	1.9 (0.1–4.9)	4.4 (1.6–11.4)	**0**.**019**
Pericarditis, in %	2.2 (1.3–3.4)	1.8 (0.1–3.4)	3.0 (1.5–5.8)	0.345
Phrenic nerve palsy, in %	2.8 (1.8–4.2)	2.7 (1.6–4.7)	2.3 (1.2–4.5)	0.843
PM implantation, in %	2.0 (1.3–3.0)	1.9 (1.3–2.8)	2.0 (0.1–4.3)	0.793

PM, pacemaker.

^a^Obtained from weighted group comparison analysis.

Bold values are significant values.

### Long-term freedom from atrial tachyarrhythmias

A total of 18 studies reported KM curves for long-term freedom from ATA, comprising 2038 patients undergoing either thoracoscopic ablation (number of studies: 10, *n* = 1189) or hybrid thoracoscopic ablation (number of studies: 8, *n* = 849). Patients undergoing hybrid thoracoscopic ablation were significantly older (62.0 vs. 58.8 years old, *P* = 0.046), had less frequent PAF history (9.6 vs. 31.1%, *P* < 0.001), and had a longer of AF history (7.0 vs. 5.3 years, *P* = 0.039) and a more advanced cardiovascular comorbidity profile compared with patients undergoing thoracoscopic ablation (*Table 3*). Additionally, patients undergoing hybrid thoracoscopic ablation underwent more frequently catheter ablation before surgical ablation compared with patients undergoing isolated thoracoscopic ablation (39.5 vs. 16.9%, *P* < 0.001) (*Table 3*).

**Table 3:** Patient and procedural characteristics of studies reporting on long-term freedom from ATA

Variables	Overall (*n* = 2 038)	Thoracoscopic (*n* = 1 189)	Hybrid (*n* = 849)	*P*-value^a^
Age, in years	60.2 (58.4–62.0)	58.8 (56.8–60.8)	62.0 (59.3–64.7)	**0**.**046**
Female subjects, in %	26.0 (22.2–30.3)	24.5 (19.9–29.6)	28.9 (22.2–36.7)	**<0**.**001**
BMI, in kg/m^2^	28.6 (27.4–28.9)	28.7 (26.4–30.9)	28.7 (27.3–30.0)	0.983
PAF history, in %	19.5 (8.1–40.0)	32.1 (11.0–64.5)	9.6 (2.6–29.1)	**<0**.**001**
AF duration, in years	6.0 (5.1–6.9)	5.3 (4.2–6.4)	7.0 (5.8–8.3)	**0**.**039**
CHA₂DS₂-VASc score	2 (2–2)	2 (1–2)	2 (2–3)	0.078
Hypertension, in %	51.2 (42.9–59.5)	46.7 (37.7–55.9)	60.0 (46.5–72.2)	**<0**.**001**
Diabetes mellitus, in %	10.8 (8.2–14.2)	10.0 (6.5–15.1)	13.1 (10.2–16.6)	**0**.**001**
Stroke, in %	10.2 (8.0–13.0)	11.0 (8.5–14.2)	8.6 (5.1–14.1)	0.570
Prior PVI, in %	27.9 (17.6–41.3)	16.9 (8.0–32.0)	39.5 (28.8–51.3)	**<0**.**001**
LVEF, in %	56.8 (54.4–59.2)	59.0 (55.6–62.3)	54.3 (51.6–57.1)	0.123
LAD, in mm	45.0 (42.7–47.4)	43.8 (41.4–46.2)	46.9 (42.5–51.4)	0.215
Procedure time, in min	215.2 (140.8–289.6)	200.2 (145.6–254.7)	233.8 (68.3–399.3)	0.690
Conversion to sternotomy, in %	1.6 (0.8–3.2)	2.1 (0.8–5.8)	1.2 (0.1–3.1)	0.131

AF, atrial fibrillation; BMI, body mass index; LAD, left atrial diameter; LVEF, left ventricular ejection fraction; PAF, paroxysmal atrial fibrillation; PM, pacemaker; PVI, pulmonary vein isolation.

^a^Obtained from unweighted groups comparison analysis.

Bold values are significant values.

**Figure 3:**
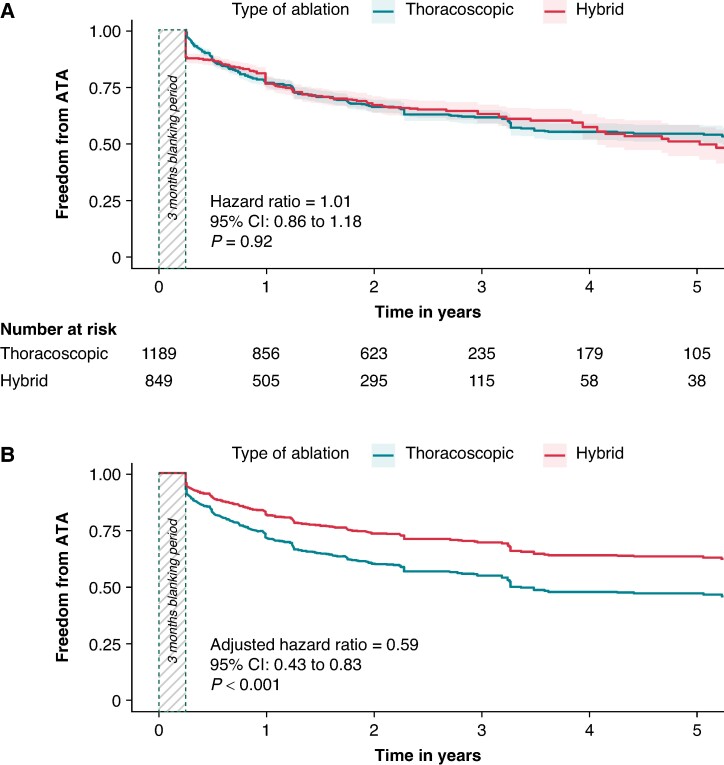
Kaplan–Meier curves for freedom from ATA for thoracoscopic and hybrid thoracoscopic AF ablation. (*A*) Unadjusted. (*B*) Adjusted for confounders including age, sex, paroxysmal AF history, duration of AF history, and frailty term (study variable). AF, atrial fibrillation; ATA, atrial tachyarrhythmia; CI, confidence interval.

For studies reporting on long-term freedom from ATA, there were no differences in short-term outcomes between patients undergoing hybrid thoracoscopic or thoracoscopic ablation (*Table 4*). Unadjusted survival analysis showed no differences in freedom from ATA between patients undergoing hybrid thoracoscopic or isolated thoracoscopic ablation (HR = 1.01, 95% CI: 0.86–1.18, *P* = 0.92) (*Figure 3A*). Because of the different risk profiles, a multivariable Cox frailty model was constructed, revealing that hybrid ablation was significantly associated with greater long-term freedom from ATA (adjusted HR = 0.59, 95% CI: 0.43–0.83, *P* < 0.001; *Figure 3B*) compared with isolated thoracoscopic ablation, after adjusting for age, sex, PAF history, duration of AF history, and frailty term (study variable) (*Table 5*). The rationale for variable selection in the adjusted model is presented in Supplementary material online, *Table S5*. Adjusted freedom from ATA at, 3, and 5 years was 71.6%, 55.1%, and 46.8% for isolated thoracoscopic ablation and 82.0%, 69.9%, and 63.6% for hybrid ablation (*P* < 0.001) (see Supplementary material online, *Table S6*).

**Table 4:** Short-term outcomes of studies reporting on long-term freedom from ATA

Variables	Overall (*n* = 2 038)	Thoracoscopic (*n* = 1 189)	Hybrid (*n* = 849)	*P*-value^a^
Early mortality, in %	0.9 (0.5–1.5)	0.6 (0.2–1.6)	1.0 (0.1–2.0)	0.065
Early stroke, in %	1.5 (0.8–2.8)	1.9 (0.8–4.5)	0.9 (0.3–1.9)	0.105
Early reoperations, in %	1.8 (1.0–3.0)	2.2 (1.4–3.5)	1.4 (0.1–3.9)	0.282
Cardiac tamponade, in %	2.5 (1.1–5.8)	3.2 (0.6–13.1)	1.9 (0.1–4.4)	0.557
Any bleeding, in %	3.5 (2.1–5.8)	2.6 (0.9–7.5)	3.1 (1.7–5.7)	0.308
Pneumonia, in %	1.8 (1.1–3.0)	1.7 (0.9–3.1)	1.9 (0.1–5.6)	0.866
Pleural effusion, in %	2.2 (1.0–4.9)	2.0 (0.7–6.0)	2.4 (0.1–8.3)	0.425
Pericarditis, in %	1.8 (0.1–3.6)	1.0 (0.5–2.4)	2.4 (1.0–5.6)	0.145
Phrenic nerve palsy, in %	1.4 (0.1–2.4)	1.7 (0.6–4.3)	1.2 (0.1–2.4)	0.940
PM implantation, in %	1.3 (0.1–2.0)	1.4 (0.8–2.4)	1.3 (0.1–2.4)	0.887

PM, pacemaker.

^a^Obtained from unweighted groups comparison analysis.

**Table 5:** Adjusted Cox proportional hazard frailty model for ATA recurrences

Variable	Beta	*P*-value	Hazard ratio	95% CI for HR	
				Lower	Upper
Age, per 1 year	0.068	**0.002**	1.07	1.03	1.12
Females, per 1% increase	−0.021	**<0**.**001**	0.98	0.97	0.99
Paroxysmal AF history, per 1% increase	0.002	0.200	1.00	0.99	1.01
AF duration, per 1-month increase	0.001	0.840	1.00	0.99	1.01
Hybrid AF ablation	−0.515	**<0**.**001**	0.59	0.43	0.83
Frailty term (study)		**0**.**037**			

AF, atrial fibrillation; CI, confidence interval; HR, hazard ratio.

Bold values are significant values.

Sensitivity analysis was conducted to assess the effect of additional epicardial lesions in patients undergoing isolated thoracoscopic ablation, which showed no significant difference (HR = 1.22, 95% CI: 0.98–1.46, *P* = 0.070) in long-term freedom from ATA as compared with patients undergoing PVI + box alone (see Supplementary material online, *Figure S1*).

### One-stage vs. two-stage hybrid thoracoscopic atrial fibrillation ablation

Sensitivity analysis showed no differences in freedom from ATA between patients undergoing one-stage or two-stage hybrid thoracoscopic ablation compared with isolated thoracoscopic ablation in a multivariable Cox frailty model with adjustments for relevant confounders (HR = 0.62, 95% CI: 0.44–0.87, *P* = 0.0054 for one-stage hybrid; HR = 0.52, 95% CI: 0.33–0.81, *P* = 0.0044 for two-stage hybrid thoracoscopic ablation) (see Supplementary material online, *Figure S2*). The rationale for variable selection in the adjusted model is presented in Supplementary material online, *Table S7*.

### Publication bias assessment

Publication bias assessment was performed for overall patient characteristics, procedural data, and short-term outcomes to assess possible under- and over-reporting (see Supplementary material online, *Table S8*). Significant publication bias was present for the duration of AF history (Beta: 4.002, *P* = 0.017), ablation procedure time (Beta: 1.210, *P* = 0.005), pre-operative stroke history (Beta: −1.343, *P* = 0.002), and conversion to sternotomy during the index procedure (Beta: −1.971, *P* = 0.007).

For short-term outcomes, significant publication bias was present for early mortality (Beta: −3.820, *P* = 0.009), any bleeding (Beta: −2.526, *P* = 0.006), and pacemaker implantation (Beta: −3.896, *P* = 0.004).

## Discussion

This study provides a comprehensive real-world assessment of isolated thoracoscopic and hybrid thoracoscopic AF ablation. The key findings of this study are as follows: (i) both isolated thoracoscopic ablation and hybrid thoracoscopic ablation are efficacious and safe procedures for AF treatment; (ii) when adjusted for clinically relevant patient characteristics and electrophysiological confounders, hybrid thoracoscopic ablation exhibits a greater long-term freedom from ATA as compared with isolated thoracoscopic ablation; (iii) when adjusted for clinically relevant patient characteristics and electrophysiological confounders, there is no difference in freedom from ATA between one-stage and two-stage hybrid thoracoscopic ablation; (iv) there is no statistically significant difference between the procedures in terms of complications; and (v) early complication rates are low for both isolated thoracoscopic and hybrid thoracoscopic ablation; however, it must be noted that there was significant publication bias suggestive for under-reporting for some of the short-term outcomes.

### Outcomes and safety of isolated and hybrid thoracoscopic atrial fibrillation ablation

For both isolated and hybrid thoracoscopic AF ablation, several RCTs show superiority in achieving freedom from ATA at 12-month follow-up compared with catheter ablation. The FAST trial showed higher ATA freedom for isolated thoracoscopic compared with catheter ablation (36.5% vs. 65.6%, *P* = 0.0022), and this difference in ATA recurrence remained significant on the long term (7 years).^3,14^ A meta-analysis of three prospective randomized trials confirmed a lower rate of incident atrial arrhythmia recurrence after thoracoscopic vs. catheter ablation.^14^ These findings stand in contrast with a recent RCT in (long-standing) persistent AF patients showing a freedom from ATA of 26% for isolated thoracoscopic ablation and 28% in the catheter group at 12-month follow-up.^15^ However, it is important to note that the poor results of 26% and 28% could partly be explained by the continuous rhythm monitoring used in the study, which provides a more accurate detection of ATA recurrences. Additionally, the CASA-AF trial utilized outdated surgical tools, had limited requirements for surgical expertise, and involved more extensive ablation in the catheter group, all of which may have influenced the comparative outcomes.

Although radiofrequency catheter ablation has shown modest efficacy in paroxysmal AF, the extent to which these benefits apply to persistent AF remains unclear, with additional validation needed. Surgical AF ablation, including both isolated and hybrid approaches, provides a viable alternative for persistent AF patients.^16^ Based on several meta-analysis, the overall success rate of a hybrid AF procedure in terms of ATA freedom varies between 59 and 88%.^5^ It is important to note that in these meta-analyses, outcomes following a subxiphoid or trans-diaphragmatic approach are reported as inferior compared with those of a thoracoscopic approach.^5^ A pooled analysis of 34 nonrandomized studies using a thoracoscopic access suggested that hybrid thoracoscopic ablation results in a freedom of ATA in patients with persistent and long-standing-persistent AF of around 70%.^6^ There are two RCTs evaluating outcomes after hybrid thoracoscopic AF ablation compared with catheter ablation in ablation-naïve patients with (long-standing) persistent AF. In the HARTCAP-AF trial, freedom from ATA off anti-arrhythmic drugs (AADs) was significantly higher in the hybrid arm compared with the catheter arm at 12 months (89% vs. 41%, *P* = 0.002).^17^ In the multicentre CEASE-AF trial, freedom from ATA recurrences or increased doses of previously failed AADs was 71.6% in the hybrid arm vs. 39.2% in the catheter arm at 12 months (*P* < 0.001).^18^

Although thoracoscopic AF ablation is a minimally invasive procedure, it remains a surgical procedure, and therefore, complications may occur more often after thoracoscopic ablation compared with less invasive procedures, such as catheter ablation.^3,15,19^ Especially noteworthy are complications such as bleeding requiring transfusion, conversion to sternotomy, phrenic nerve injury, cardiac tamponade, and pneumothorax, which seem to occur more frequently after isolated thoracoscopic than after catheter ablation, as reported in the literature.^3,15,19^ Nevertheless, these complications are relatively infrequent after isolated and hybrid thoracoscopic AF ablation,^6^ and in the recent HARTCAP-AF and the CEASE-AF trial, no differences in safety outcomes were reported between the hybrid and the catheter arm.^17,18^

### Outcomes and safety of isolated compared with hybrid thoracoscopic atrial fibrillation ablation

The current meta-analysis distinguishes itself through its comprehensive methodology, encompassing data from diverse patient cohorts across various international centres, including data reporting both isolated thoracoscopic and hybrid thoracoscopic AF ablation, as well as those exclusively analysing either procedure, providing a real-world overview of the surgical treatment of lone AF. By synthesizing multiple KM curves into a unified curve, this analysis offers nuanced insights into the comparative efficacy and safety profiles of both procedures, in addition to their determinants. Of note, because of the differences in techniques between a hybrid ablation via a thoracoscopic vs. a subxiphoid or trans-diaphragmatic approach and the lower success rates in the latter,^5^ the current analysis focuses solely on studies evaluating hybrid thoracoscopic ablation. No difference was found in unadjusted freedom from ATA between patients undergoing hybrid thoracoscopic or isolated thoracoscopic ablation. However, patients undergoing hybrid thoracoscopic ablation had a more advanced cardiovascular comorbidity profile compared with those undergoing isolated thoracoscopic ablation and a more advanced AF substrate, which was reflected by longer duration of AF history and more frequently reported history of previous catheter ablation. The potential impact of the latter on the long-term freedom from ATA might be marginal due to the more antral location of the PVI lesions acquired after thoracoscopic ablation as compared with catheter ablation. To address these differences between patient characteristics, adjustments for various confounding factors were performed. The adjusted analysis showed higher success rates for hybrid thoracoscopic ablation compared with isolated thoracoscopic ablation.

The long-term benefit of hybrid ablation can be attributed to several factors. Firstly, hybrid thoracoscopic ablation includes endocardial validation of the box lesion, which ensures that isolation remains effective over time. Without this thorough validation, there is a risk that gaps in the box lesion may lead to recurrences of atrial arrhythmias despite initial success. Additionally, hybrid ablation addresses other arrhythmias that the patient may have or that may develop during the procedure, contributing to improved long-term freedom from ATA. This comprehensive approach helps to reduce the likelihood of arrhythmia recurrences and enhances overall treatment efficacy.

Often, the combination of thoracoscopic AF ablation with catheter ablation is believed to increase the complication rate. In theory, the combination of these procedures indeed could add up the complications of each individual procedure. Still, a complication can only occur once and the combination of both procedures can also be protective (e.g. opening the pericardium during thoracoscopy will prevent the risk of phrenic nerve injury during catheter ablation). Our current analysis did not show differences in safety outcomes between isolated and hybrid thoracoscopic AF ablation. Nevertheless, there was significant publication bias suggestive for under-reporting for some of the short-term outcomes across the literature.

A previous meta-analysis, comparing these two approaches, concluded there were no differences in freedom from ATA at 12- and 24-month follow-up between hybrid thoracoscopic ablation and isolated thoracoscopic ablation [thoracoscopic alone 71.5% (95% CI 66.1–76.9), hybrid 63.2% (95% CI 51.5–75.0), thoracoscopic alone 68.5% (95% CI 57.7–79.3), and hybrid 57.0% (95% CI 33.6–80.4), respectively].^7^ However, this analysis was based on a limited dataset, primarily consisting of case series, with a maximum follow-up of (only) 24 months. The distinctiveness of the current meta-analysis lies in its ability to synthesize evidence from a considerably diverse and larger patient cohort, incorporating studies across various centres and continents. This comprehensive analysis allows for a more refined assessment of the comparative effectiveness of hybrid thoracoscopic and isolated thoracoscopic ablation in achieving freedom from ATA. Furthermore, freedom from ATA was not only assessed at a longitudinal time point but also as a time-to-event outcome, which is generally considered to have more power while providing insight into the timing of recurrences.

Additionally, no significant difference was observed in long-term freedom from ATA between one-stage and two-stage hybrid ablation procedures. This finding suggests that both approaches are equally effective in achieving sustained arrhythmia control, providing flexibility in clinical decision-making based on patient-specific factors and logistical considerations. The lack of difference also indicates that the timing of endocardial validation may not critically impact the overall effectiveness, allowing for individualized treatment without compromising efficacy.

While the success rates of both isolated thoracoscopic and hybrid thoracoscopic AF ablations are favourable, several studies reporting on hybrid thoracoscopic ablation show conduction gaps following solely epicardial ablation.^17,20,21^ The HARTCAP-AF trial demonstrated that ∼42% of patients required an endocardial touch-up due to gaps in the lesions created by epicardial ablation.^17^ The necessity to complement an epicardial procedure with an endocardial approach underscores the potential benefits of a hybrid AF ablation in optimizing long-term AF outcomes. The integration of both epicardial and endocardial ablation approaches in hybrid thoracoscopic ablation potentially explains the increased efficacy in addressing AF recurrence and offering a more comprehensive treatment approach.

### Limitations

While the current study possesses several strengths, such as a large number of included studies and adjustments for clinical confounders, there are some important limitations that should be addressed. Firstly, like all meta-analyses, the findings of this study are influenced by the quality and methodology of the included studies. This limitation affects both the study selection process and the quality of follow-up data, including variations in ATA recording methods. Nevertheless, the study quality has been carefully assessed and found to be of overall high quality. Secondly, pooling data from multiple studies might introduce heterogeneity to the results of the meta-analyses, primarily due to differences in patient characteristics. An attempt was made to correct for clinical heterogeneity by performing adjusted analyses with an additional correction for study frailty, enabling the assessment of residual unexplained heterogeneity. The analysis for long-term freedom from ATA showed significant frailty, suggesting the presence of residual heterogeneity. Thirdly, significant publication bias was identified, suggesting under-reporting of short-term adverse events, such as early mortality, early bleedings, and pacemaker implantation. This bias may arise due to a tendency for higher-volume centres to be more inclined to publish their results, while centres with suboptimal outcomes might not report their results as frequently. Fourthly, this study is limited by the absence of comparative data directly evaluating both isolated thoracoscopic and hybrid thoracoscopic AF ablation. However, to date, there is a lack of alternative studies comparing both procedures, constraining a more extensive comparative analysis. Lastly, due to insufficient data in the included studies reporting on post-operative antiarrhythmic therapy, it was not feasible to adjust for differences in treatment strategies.

## Conclusion

In this meta-analysis, hybrid thoracoscopic AF ablation is associated with greater long-term freedom from ATA when compared with isolated thoracoscopic ablation, without significantly increasing early complication rates, despite evidence for overall under-reporting of short-term adverse events in the literature. There was no difference in freedom of ATA between one-stage and two-stage hybrid thoracoscopic AF ablation. However, there is a need for further high-quality research, particularly RCTs, to more comprehensively evaluate the effectiveness of both procedures, especially regarding long-term freedom from ATA.

## Supplementary Material

euae232_Supplementary_Data

## Data Availability

The data underlying this article are available in the article and in its online supplementary material.
