# Supplementary material for: Short- and long-term outcomes in isolated vs. hybrid thoracoscopic ablation in patients with atrial fibrillation: a systematic review and reconstructed individual patient data meta-analysis
Source: Europace. 2024 Sep 10;26(10):euae232. doi: 10.1093/europace/euae232 (PMC11448334; doi:10.1093/europace/euae232)
Supplement: euae232_Supplementary_Data [file euae232_supplementary_data.zip › Revision 1 Supplementary Materials.docx]

**Supplementary material**

**Short- and long-term outcomes in thoracoscopic versus hybrid thoracoscopic ablation in patients with atrial fibrillation: a systematic review and reconstructed individual patient data meta-analysis.**

Luca Aerts, MD^*^, Michal J. Kawczynski, MD^*^, Elham Bidar, MD, PhD, Justin Luermans, MD, PhD, Marisevi Chaldoupi, MD, PhD, Mark La Meir, MD, PhD, Mariusz Kowaleski, MD, PhD, Jos G. Maessen, MD, PhD, Samuel Heuts, MD, PhD, Bart Maesen, MD, PhD

Content

Supplementary Material 1 – Search strategy in electronic databases page 2

Supplementary Table 1 – Data collection worksheet page 5

Supplementary Table 2 – Study characteristics page 7

Supplementary Table 3 – Study quality assessment page 12

Supplementary Table 4 – Procedural characteristics page 14

Supplementary Table 5 – Rationale for Covariate Selection page 18

Supplementary Table 6 – Long-term freedom from ATA (adjusted) page 19

Supplementary Table 7 – Sensitivity analysis hybrid ablation page 20

Supplementary Table 8 – Publication bias assessment page 21

Supplementary Figure 1 – Effect of additional lesions on long-term ATA page 22

Supplementary Figure 2 – Long-term freedom from ATA hybrid ablation page 23

Supplementary Material 2 – References page 24

**Supplementary Material 1.** Search in electronic databases including PubMed, EMBASE, and Cochrane Library

| Overview | | |
| --- | --- | --- |
| Database | MEDLINE & PubMed Central | |
| Platform | PubMed | |
| Date of search | September 5^th^, 2023 | |
| Number of results | 12,588 | |
| Syntax guide | | |
| [MeSH] | Medical subject headings | |
| Free terms | Search performed with “All fields” | |
| Search | Query | Items found |
| #1 | Maze Procedure[MeSH] | 71 |
| #2 | Thoracoscopic ablation | 575 |
| #3 | Thoracoscopic AF ablation | 216 |
| #4 | Totally Thoracoscopic ablation | 58 |
| #5 | TT Maze | 27 |
| #6 | Minimally invasive ablation | 5,638 |
| #7 | Minimally invasive surgical ablation | 4,660 |
| #8 | VATS ablation | 231 |
| #9 | Video assisted Thoracoscopic ablation | 134 |
| #10 | Hybrid AF ablation | 263 |
| #11 | Surgical ablation | 76,161 |
| #12 | Totally Thoracoscopic MAZE | 15 |
| #13 | Thoracoscopic MAZE | 84 |
| #14 | Mini MAZE | 187 |
| #15 | Hybrid ablation | 2,539 |
| #16 | Minimally invasive AF ablation | 239 |
| #17 | Minimally invasive atrial fibrillation ablation | 469 |
| #18 | Endoscopic ablation | 7,163 |
| #19 | Endoscopic AF ablation | 274 |
| #20 | Endoscopic atrial fibrillation | 1,134 |
| #21 | #1 OR #2 OR #3 OR #4 OR #5 OR #6 OR #7 OR #8 OR #9 OR #10 OR #11 OR #12 OR #13 OR #14 OR #15 OR #16 OR #17 OR #18 OR #19 OR #20 | 80,309 |
| #22 | atrial fibrillation[MeSH Terms] | 71,605 |
| #23 | AF | 105,080 |
| #24 | Atrial fibrillation | 108,068 |
| #25 | Lone AF | 662 |
| #26 | atrial tachyarrhythmia | 21,525 |
| #27 | supraventricular tachyarrhythmia | 12,850 |
| #28 | Afib | 108,144 |
| #29 | Longstanding AF | 793 |
| #30 | Longstanding persistent AF | 662 |
| #31 | Paroxysmal atrial fibrillation | 108,068 |
| #32 | Paroxysmal AF | 108,121 |
| #33 | #22 OR #23 OR #24 OR #25 OR #26 OR #27 OR #28 OR #29 OR #30 OR #31 OR #32 | 192,629 |
| #34 | #21 AND #33 | 20,2271 |
| #35 | Publication date filter 01.01.2012 till 05.09.2023 | 12,588 |

| Overview | | |
| --- | --- | --- |
| Database | Embase library including MEDLINE | |
| Platform | Embase | |
| Date of search | September 5^th^, 2023 | |
| Number of results | 1,965 | |
| Syntax guide | | |
| Free terms | Search performed with “All fields” | |
| Search | Query | Items found |
| #1 | Maze Procedure.af | 1,999 |
| #2 | Thoracoscopic ablation.af | 203 |
| #3 | Thoracoscopic AF ablation.af | 37 |
| #4 | Totally Thoracoscopic ablation.af | 37 |
| #5 | TT Maze.af | 7 |
| #6 | Minimally invasive ablation.af | 133 |
| #7 | Minimally invasive surgical ablation.af | 78 |
| #8 | VATS ablation.af | 4 |
| #9 | Video assisted Thoracoscopic ablation.af | 3 |
| #10 | Hybrid AF ablation.af | 67 |
| #11 | Surgical ablation.af | 2,700 |
| #12 | Totally Thoracoscopic MAZE.af | 15 |
| #13 | Thoracoscopic MAZE.af | 35 |
| #14 | Mini MAZE.af | 99 |
| #15 | Hybrid ablation.af | 277 |
| #16 | Minimally invasive AF ablation.af | 1 |
| #17 | Minimally invasive atrial fibrillation ablation.af | 3 |
| #18 | Endoscopic ablation.af | 678 |
| #19 | Endoscopic AF ablation.af | 3 |
| #20 | Endoscopic atrial fibrillation.af | 0 |
| #21 | #1 OR #2 OR #3 OR #4 OR #5 OR #6 OR #7 OR #8 OR #9 OR #10 OR #11 OR #12 OR #13 OR #14 OR #15 OR #16 OR #17 OR #18 OR #19 OR #20 | 5,604 |
| #22 | atrial fibrillation.af | 202,334 |
| #23 | AF.af | 185,807 |
| #24 | Lone AF.af | 860 |
| #25 | atrial tachyarrhythmia.af | 1,544 |
| #26 | supraventricular tachyarrhythmia.af | 481 |
| #27 | Afib.af | 1,866 |
| #28 | Longstanding AF.af | 81 |
| #29 | Longstanding persistent AF.af | 349 |
| #30 | Paroxysmal atrial fibrillation.af | 15,606 |
| #31 | Paroxysmal AF.af | 9,772 |
| #32 | #22 OR #23 OR #24 OR #25 OR #26 OR #27 OR #28 OR #29 OR #30 OR #31 | 316,290 |
| #33 | #21 AND #32 | 2,985 |
| #34 | Publication date filter 2012 till 2024 | 1,965 |

| Overview | | |
| --- | --- | --- |
| Database | Cochrane Library | |
| Platform | Cochrane Library | |
| Date of search | September 5^th^, 2023 | |
| Number of results | 402 | |
| Syntax guide | | |
| [MeSH] | Medical subject heading descriptor | |
| Free terms | Search performed with “All fields” | |
| Search | Query | Items found |
| #1 | Maze Procedure[MeSH] | 5 |
| #2 | Thoracoscopic ablation | 94 |
| #3 | Thoracoscopic AF ablation | 67 |
| #4 | Totally Thoracoscopic ablation | 6 |
| #5 | TT Maze | 3 |
| #6 | Minimally invasive ablation | 517 |
| #7 | Minimally invasive surgical ablation | 283 |
| #8 | VATS ablation | 13 |
| #9 | Video assisted Thoracoscopic ablation | 25 |
| #10 | Hybrid AF ablation | 49 |
| #11 | Surgical ablation | 2,158 |
| #12 | Totally Thoracoscopic MAZE | 2 |
| #13 | Thoracoscopic MAZE | 5 |
| #14 | Mini MAZE | 33 |
| #15 | Hybrid ablation | 115 |
| #16 | Minimally invasive AF ablation | 39 |
| #17 | Minimally invasive atrial fibrillation ablation | 46 |
| #18 | Endoscopic ablation | 525 |
| #19 | Endoscopic AF ablation | 34 |
| #20 | Endoscopic atrial fibrillation | 70 |
| #21 | #1 OR #2 OR #3 OR #4 OR #5 OR #6 OR #7 OR #8 OR #9 OR #10 OR #11 OR #12 OR #13 OR #14 OR #15 OR #16 OR #17 OR #18 OR #19 OR #20 | 2,863 |
| #22 | atrial fibrillation[MeSH Terms] | 7,431 |
| #23 | AF | 17,477 |
| #24 | Atrial fibrillation | 16,016 |
| #25 | Lone AF | 53 |
| #26 | atrial tachyarrhythmia | 381 |
| #27 | supraventricular tachyarrhythmia | 187 |
| #28 | Afib | 126 |
| #29 | Longstanding AF | 231 |
| #30 | Longstanding persistent AF | 191 |
| #31 | Paroxysmal atrial fibrillation | 2,494 |
| #32 | Paroxysmal AF | 1,795 |
| #33 | #22 OR #23 OR #24 OR #25 OR #26 OR #27 OR #28 OR #29 OR #30 OR #31 OR #32 | 26,438 |
| #34 | #21 AND #33 | 446 |
| #35 | Publication date filter 01.01.2012 till 05.09.2023 | 402 |

| **Supplementary Table 1.** Predefined data collection worksheet. | |
| --- | --- |
| **Parameters** | **Units** |
| Study name | NA |
| Study author | NA |
| Year of publication | Year |
| Continent | NA |
| Country | NA |
| City | NA |
| Centre | NA |
| Patients | N |
| Inclusion period | Years |
| Age (mean, SD) | Years |
| Male sex | N, % |
| Body mass index (mean, SD) | kg/m^2^ |
| CHA2DS2-VASc score (mean, SD) | NA |
| Heart failure | N, % |
| Hypertension | N, % |
| Peripheral arterial disease | N, % |
| Diabetes mellitus | N, % |
| Prior stroke | N, % |
| Coronary Artery Disease | N, % |
| Hyperlipedemia | N, % |
| Chronic obstructive pulmonary disease | N, % |
| Previous cardiac surgery | N, % |
| Pacemaker implantation | N, % |
| Paroxysmal artrial fibrillation | N, % |
| Persistent atrial fibrillation | N, % |
| Long standing persistent atrial fibrillation | N, % |
| Duration atrial fibrillation (mean, SD) | Months |
| Prior catheter ablation | N, % |
| Oral anticoagulant | N, % |
| Antiarrhythmic drugs | N, % |
| Antiarrhythmic drugs class I | N, % |
| Antiarrhythmic drugs class II | N, % |
| Antiarrhythmic drugs class III | N, % |
| LVEF (mean, SD) | % |
| Left atrial volume indexed (mean, SD) | % |
| Left atrial diameter (mean, SD) | % |
| Any mitral regurgitation | N, % |
| Hybrid | N, % |
| Hybrid 1-stage | N, % |
| Hybrid 2-staged | N, % |
| Thoracoscopic | N, % |
| Thoracoscopic, right-sided | N, % |
| Thoracoscopic, left-sided | N, % |
| Thoracoscopic, bilateral | N, % |
| LAA closure | N, % |
| Conversion rate | N, % |
| Procedural time (mean, SD) | Minutes |
| ICU stay (mean, SD) | Days |
| Hospital stay (mean, SD) | Days |
| Mortality | N, % |
| Reintubation | N, % |
| Myocardial infarction | N, % |
| Stroke | N, % |
| Rethoracotomy | N, % |
| Tamponade | N, % |
| Bleeding | N, % |
| Pneumonia | N, % |
| Pneumothorax | N, % |
| Pleural effusion | N, % |
| Pericarditis | N, % |
| Phrenic nerve palsy | N, % |
| Pacemaker implantation | N, % |
| Pulmonary vein stenosis | N, % |
| Reporting on freedom from atrial tachyarrhythmias using Kaplan-Meier curves | Yes/no |
| *Abbreviations: LVEF: left ventricular ejection fraction, NA: not applicable, SD: standard deviation.* | |

| **Supplementary Table 2. Study characteristics** | | | | | | |
| --- | --- | --- | --- | --- | --- | --- |
| **Study** | **Publication year** | **Prospective vs. retrospective** | **Cohort size (N=)** | **Hybrid vs. thoracoscopic** | **FU duration^a^** | **Monitoring & definition of ATA recurrences** |
| Al-Jazairi M^1^ | 2019 | Prospective | 50 | Hybrid | 1 year | 72-h Holter at 3,6, and 12 months. Any duration of ATA |
| An K^2^ | 2018 | Prospective | 60 | Thoracoscopic | 2 years | 24-h Holter at 3, 6, 12, and 24 months. >30sec of ATA |
| Arhire A^3^ | 2009 | Retrospective | 16 | Hybrid | 6 months | 24-h or 6-days Holter at 6 months (further unspecified) |
| Baretti R^4^ | 2015 | Retrospective | 76 | Thoracoscopic | 2 years | Every 3 months Holter. Any duration of ATA |
| D’Alessandro C^5^ | 2020 | Retrospective | 21 | Thoracoscopic | 10 to 4 months | *NR* |
| De Maat G^6^ | 2014 | Prospective | 33 | Thoracoscopic | 12.6±2 months | 96-h Holter at 6 months and 24-h at 12 months. >30sec of ATA |
| Doty J^7^ | 2012 | Prospective | 32 | Thoracoscopic | 28 (range: 4-43) months | 12-lead ECG at 1, 3, 6, and 12 months |
| Driessen A^8^ | 2017 | Prospective | 66 | Thoracoscopic | 66 (range: 60-82) months | 3-monthly 24-h Holter for 2 years. 2-5 years ECG’s. >30sec of ATA |
| Dunnington G^9^ | 2021 | Retrospective | 455 | Hybrid | Maximum reported: 36 months | 12-lead ECG at 2 weeks, 2 months and annually. Also patients with ILR. Any ATA. |
| Edgerton J^10^ | 2007 | Retrospective | 83 | Thoracoscopic | 6 months | 12-lead ECG at 1, 3, and 6 months. Holter at 6 months if possible. >15sec of ATA |
| Gasbarri T^11^ | 2014 | Retrospective | 24 | Hybrid | 15.4±8 months | 24-h Holter at 3-6, 9, 12, 18, and 24 months. |
| Geuzebroek G^12^ | 2016 | Prospective | 82 | Thoracoscopic | 11.3±5.6 months | 24-h Holter at 3, 6, and 12 months. |
| Guo H^13^ | 2019 | Retrospective | 54 | Hybrid | 14 (range: 3-30) months | 24-h Holter at 3, 6, and 12 months. >30sec of ATA |
| Haldar S^14^ | 2017 | Prospective | 60 | Thoracoscopic | 12 months | 7-day Holter at 3, 6, 9, and 12 months. >30sec of ATA |
| Harlaar N^15^ (Leiden) | 2020 | Prospective | 42 | Thoracoscopic | 12 months | 24-h Holter at 3, 6, and 12 months. >30sec of ATA |
| Harlaar N^15^ (Eindhoven) | 2020 | Prospective | 38 | Thoracoscopic | 12 months | 24-h Holter at 3, 6, and 12 months. >30sec of ATA |
| Janusauskas V^16^ | 2016 | Retrospective | 91 | Thoracoscopic | 5 (range: 1-7) years | 24-h Holter at 3, 6, and 12 months. Thereafter annual 24-h Holter. >30sec of ATA |
| Ji Y^17^ | 2021 | Prospective | 31 | Thoracoscopic | 5 years | 24-h Holter at 3, 6, and 12 months. Thereafter 24-h Holter at 3 and 5 years. >30sec of ATA |
| Khoynezhad A^18^ | 2015 | Retrospective | 48 | Thoracoscopic | *NR* | *NR* |
| Kim J^19^ | 2021 | Retrospective | 306 | Thoracoscopic | Mean 30 months | 24-h Holter at 3, 6, and 12 months. Thereafter annual 24-h Holter. |
| Lapenna E^20^ | 2021 | Retrospective | 50 | Hybrid | 2 years | 12-lead ECG at 3, 6, 12, 18, and 24 months. Also ILR implantation in some patients (not further specified) |
| Maesen B^21^ | 2018 | Retrospective | 64 | Hybrid | 1732 (range: 1084-2481) days | 7-day Holter at 3, 6, 9, 12, 24, and 36 months. >30sec of ATA |
| Mahapatra S^22^ | 2011 | Prospective | 15 | Hybrid | 20.7±4.5 months | 7-day Holter at 3, 6, and 12 months. >30sec of ATA |
| Mei J^23^ | 2014 | Prospective | 55 | Thoracoscopic | 12.6±2.2 months | 24-h Holter at 1, 3, 6, and 12 months. >30sec of ATA |
| Montero J^24^ | 2020 | Retrospective | 26 | Thoracoscopic | 1-year | *NR* |
| Nasso G^25^ | 2021 | Retrospective | 609 | Thoracoscopic | 72 (range: 2-152) months | FU at 6, 9, and 12 moths. Further unspecified |
| Neefs J^26^ | 2022 | Prospective | 498 | Thoracoscopic | 2 years | 24-h Holter monitoring at 3, 6, 9, 12, 15, 18 and 24 months. >30sec of ATA |
| Pannone L^27^ | 2023 | Retrospective | 120 | Hybrid | 62.3±20.3 months | 7-day Holter at 3,6, and 12 months. Thereafter annual 24-h Holter. >30sec of ATA |
| Pearman C^28^ | 2019 | Retrospective | 35 | Thoracoscopic | 17.7±7.4 months | 12-lead ECG at 3, 6, and 12 months. Holter FU at physician’s discretion. |
| Pesl M^29^ | 2022 | Retrospective | 52 | Hybrid | 12 months | 24-h Holter at 1, 3, 6, and 12 months. >30sec of ATA |
| Pick A^30^ | 2023 | Retrospective | 48 | Hybrid | 1054 (range: 26-1719) days | 24-h Holter at 6 and 12 months. >30sec of ATA |
| Pojar M^31^ | 2019 | Retrospective | 65 | Thoracoscopic | 866 (IQR: 612-1185) days | 24-h Holter at 3, 6, and 12 months. >30sec of ATA |
| Pokushalov E^32^ | 2014 | Prospective | 60 | Thoracoscopic | 12 months | Reveal XT implantation in all patients. Any ATA |
| Pong T^33^ | 2022 | Prospective | 84 | Hybrid | 18.7±13 months | 14 days Holter at 3, 6, and 12 months. >30sec of ATA |
| Probst J^34^ | 2016 | Prospective | 60 | Thoracoscopic | 12 months | 24-h Holter at 6 and 12 months. >30sec of ATA |
| Pruitt J^35^ | 2007 | Retrospective | 100 | Hybrid | Mean 23.1 months | ECG or Holter monitoring, not prespecified |
| Richardson T^36^ | 2015 | Retrospective | 83 | Hybrid | 12 months | ECG or Holter monitoring, not prespecified. >30sec of ATA |
| Rodrigues C^37^ | 2021 | Retrospective | 15 | Thoracoscopic | 12 months | 24-h Holter at 6 months. At 12 and 18 months external recorder for 7 days |
| Rollin A^38^ | 2020 | Prospective | 15 | Hybrid | 9±8 months | Holter at 6 and 12 months (unknown duration). >30sec of ATA |
| Saini A^39^ | 2017 | Prospective | 109 | Thoracoscopic | 5 years (37.8% complete | Ambulatory monitoring at 6, 12, 24, 36, 48, and 60 months (unknown duration). >30sec of ATA |
| Santini M^40^ | 2012 | Retrospective | 22 | Thoracoscopic | 22 (IQR: 20-27) months | 24-h Holter at 6, 9, and 12 months. Thereafter every 6 months. |
| Schenk S^41^ | 2015 | Retrospective | 42 | Thoracoscopic | *NR* | 12-lead ECG’s, Holter and implantable devices (not further specified) |
| Sirak J^42^ | 2012 | Retrospective | 229 | Thoracoscopic | 24 months | 7-day Holter at 3, 6, 13, and 24 months. >30sec of ATA |
| Suwalski P^43^ | 2011 | Retrospective | 45 | Hybrid | 30.2 (range: 2-48) months | Holter at 3, 6, 12, 24, and 48 months. |
| Tan C^44^ | 2021 | Prospective | 50 | Hybrid | 29±13 months | 7-day Holter at 3 and 6 months. Thereafter every 6 months. >30sec of ATA |
| Troubil M^45^ | 2022 | Retrospective | 79 | Thoracoscopic | 3.1±1.4 years | 7-day Holter at FU visit (timing unknown). >30sec of ATA |
| Van Der Heijden C^46^ | 2022 | Retrospective | 154 | Hybrid | 2 years | Holter at 3, 12, and 24 months. >30sec of ATA |
| Wang J^47^ | 2011 | Retrospective | 91 | Thoracoscopic | 2.2 (range: 1.0-3.6) years | 24-48-h Holter at 1, 3, 6, and 12 months. >30sec of ATA |
| Witkowska A^48^ | 2015 | Retrospective | 47 | Hybrid | *NR* | 48-h Holter at 3, 6, and 12 months. |
| Yan T^49^ | 2022 | Retrospective | 102 | Thoracoscopic | 22.8±13.9 months | 24-h Holter at 3, 6, and 12 months. Thereafter every 12 months |
| Yu C^50^ | 2021 | Prospective | 91 | Thoracoscopic | 5 years | 24-h Holter at 3, 6, and 12 months. Thereafter every 12 months. >30sec of ATA |
| Zheng S^51^ | 2013 | Prospective | 139 | Thoracoscopic | 55 (range: 3-73) months | 24-h Holter at 1, 3, 6, and 12 months. |
| Zotov A^52^ | 2020 | Retrospective | 28 | Thoracoscopic | 9 (range: 6-16) months | 24-h Holter at 3 and 6 months. >30sec of ATA |
| Abbreviations: ATA: atrial tachyarrhythmia, IQR: interquartile range. | | | | | | |

| **Supplementary Table 3. Study quality assessment** | | | | | | | | | | | |
| --- | --- | --- | --- | --- | --- | --- | --- | --- | --- | --- | --- |
| **Author** | **Study arms** | **Selection** | | | | **Comparability** | **Outcome** | | | **Overall grade** | **Remark** |
| Al-Jazairi M^1^ | 2 | 1 | 1 | 1 | 1 | 2 | 1 | 1 | 1 | 9/9 |  |
| An K^2^ | 1 | 1 | 1 | 1 | 1 | 0 | 1 | 1 | 1 | 7/9 |  |
| Arhire A^3^ | 1 | 1 | 1 | 1 | 1 | 0 | 1 | 0 | 0 | 5/9 | Conference |
| Baretti R^4^ | 1 | 1 | 1 | 1 | 1 | 0 | 1 | 1 | 1 | 7/9 | Conference |
| D’Alessandro C^5^ | 2 | 1 | 1 | 1 | 1 | 0 | 0 | 1 | 0 | 5/9 | Conference |
| De Maat G^6^ | 2 | 1 | 1 | 1 | 1 | 2 | 1 | 1 | 1 | 9/9 |  |
| Doty J^7^ | 1 | 1 | 1 | 1 | 1 | 0 | 1 | 1 | 1 | 7/9 |  |
| Driessen A^8^ | 1 | 1 | 1 | 1 | 1 | 1 | 1 | 1 | 1 | 8/9 |  |
| Dunnington G^9^ | 2 | 1 | 1 | 1 | 1 | 2 | 1 | 1 | 1 | 9/9 |  |
| Edgerton J^10^ | 1 | 1 | 1 | 1 | 1 | 0 | 1 | 0 | 0 | 5/9 |  |
| Gasbarri T^11^ | 1 | 1 | 1 | 1 | 1 | 0 | 1 | 1 | 1 | 7/9 | Conference |
| Geuzebroek G^12^ | 1 | 1 | 1 | 1 | 1 | 1 | 1 | 1 | 1 | 8/9 |  |
| Guo H^13^ | 1 | 1 | 1 | 1 | 1 | 1 | 1 | 1 | 1 | 8/9 |  |
| Haldar S^14^ | 2 | 1 | 1 | 1 | 1 | 2 | 1 | 1 | 1 | 9/9 |  |
| Harlaar N^15^ | 1 | 1 | 1 | 1 | 1 | 2 | 1 | 1 | 1 | 9/9 | Leiden |
| Harlaar N^15^ | 1 | 1 | 1 | 1 | 1 | 2 | 1 | 1 | 1 | 9/9 | Eindhoven |
| Janusauskas V^16^ | 1 | 1 | 1 | 1 | 1 | 2 | 1 | 1 | 1 | 9/9 |  |
| Ji Y^17^ | 1 | 1 | 1 | 1 | 1 | 0 | 1 | 1 | 1 | 7/9 |  |
| Khoynezhad A^18^ | 1 | 0 | 1 | 1 | 1 | 0 | 0 | 0 | 0 | 3/9 | Conference |
| Kim J^19^ | 2 | 0 | 1 | 1 | 1 | 1 | 1 | 1 | 1 | 7/9 |  |
| Lapenna E^20^ | 1 | 1 | 1 | 1 | 1 | 2 | 1 | 1 | 1 | 9/9 |  |
| Maesen B^21^ | 1 | 1 | 1 | 1 | 1 | 2 | 1 | 1 | 1 | 9/9 |  |
| Mahapatra S^22^ | 2 | 1 | 1 | 1 | 1 | 1 | 1 | 1 | 1 | 8/9 |  |
| Mei J^23^ | 1 | 1 | 1 | 1 | 1 | 1 | 1 | 1 | 1 | 8/9 |  |
| Montero J^24^ | 1 | 1 | 1 | 1 | 1 | 0 | 1 | 0 | 0 | 5/9 | Conference |
| Nasso G^25^ | 1 | 1 | 1 | 1 | 1 | 0 | 0 | 1 | 0 | 5/9 |  |
| Neefs J^26^ | 1 | 1 | 1 | 1 | 1 | 2 | 1 | 1 | 1 | 9/9 |  |
| Pannone L^27^ | 1 | 1 | 1 | 1 | 1 | 2 | 1 | 1 | 1 | 9/9 |  |
| Pearman C^28^ | 2 | 1 | 1 | 1 | 1 | 2 | 0 | 1 | 0 | 7/9 |  |
| Pesl M^29^ | 1 | 1 | 1 | 1 | 1 | 0 | 1 | 1 | 1 | 7/9 |  |
| Pick A^30^ | 1 | 1 | 1 | 1 | 1 | 0 | 1 | 1 | 1 | 7/9 |  |
| Pojar M^31^ | 2 | 1 | 1 | 1 | 1 | 2 | 1 | 1 | 1 | 9/9 |  |
| Pokushalov E^32^ | 1 | 1 | 1 | 1 | - | - | 1 | 1 | 1 | - | Conference |
| Pong T^33^ | 1 | 1 | 1 | 1 | 1 | 2 | 1 | 1 | 1 | 9/9 |  |
| Probst J^34^ | 1 | 1 | 1 | 1 | 1 | 2 | 1 | 1 | 1 | 9/9 |  |
| Pruitt J^35^ | 1 | 1 | 1 | 1 | 1 | 1 | 1 | 0 | 0 | 6/9 |  |
| Richardson T^36^ | 1 | 1 | 1 | 1 | 1 | 1 | 0 | 1 | 1 | 7/9 |  |
| Rodrigues C^37^ | 1 | 1 | 1 | 1 | 1 | 0 | 1 | 1 | 0 | 6/9 |  |
| Rollin A^38^ | 1 | 1 | 1 | 1 | 1 | 1 | 1 | 1 | 1 | 8/9 |  |
| Saini A^39^ | 1 | 1 | 1 | 1 | 1 | 2 | 1 | 1 | 1 | 9/9 |  |
| Santini M^40^ | 1 | 1 | 1 | 1 | 1 | 1 | 1 | 1 | 1 | 8/9 |  |
| Schenk S^41^ | 1 | 1 | 1 | 1 | 1 | 1 | 0 | 0 | 0 | 5/9 | Conference |
| Sirak J^42^ | 1 | 1 | 1 | 1 | 1 | 0 | 1 | 1 | 0 | 6/9 |  |
| Suwalski P^43^ | 1 | 1 | 1 | 1 | 1 | 0 | 1 | 1 | 0 | 6/9 | Conference |
| Tan C^44^ | 1 | 1 | 1 | 1 | 1 | 1 | 1 | 1 | 1 | 8/9 |  |
| Troubil M^45^ | 1 | 1 | 1 | 1 | 1 | 0 | 0 | 1 | 1 | 6/9 |  |
| Van Der Heijden C^46^ | 1 | 1 | 1 | 1 | 1 | 2 | 1 | 1 | 1 | 9/9 |  |
| Wang J^47^ | 2 | 1 | 1 | 1 | 1 | 1 | 1 | 1 | 1 | 8/9 |  |
| Witkowska A^48^ | 1 | 1 | 1 | 1 | 1 | - | 1 | 1 | 0 | - | Conference |
| Yan T^49^ | 1 | 1 | 1 | 1 | 1 | 1 | 1 | 1 | 1 | 8/9 |  |
| Yu C^50^ | 1 | 1 | 1 | 1 | 1 | 2 | 1 | 1 | 1 | 9/9 |  |
| Zheng S^51^ | 1 | 1 | 1 | 1 | 1 | 2 | 1 | 1 | 1 | 9/9 |  |
| Zotov A^52^ | 1 | 1 | 1 | 1 | 1 | 0 | 1 | 0 | 0 | 5/9 |  |

| **Supplementary Table 4. Procedure characteristics** | | | |
| --- | --- | --- | --- |
| **Study** | **Hybrid vs. thoracoscopic** | **Lesion Set** | **LAA management** |
| Al-Jazairi M^1^ | Hybrid | PVI + box + epicardial lesions + endocardial lesions | Clip |
| An K^2^ | Thoracoscopic | PVI + box + epicardial lesions | Stapler |
| Arhire A^3^ | Hybrid | PVI + box + endocardial validation | *NR* |
| Baretti R^4^ | Thoracoscopic | PVI + box | Suture |
| D’Alessandro C^5^ | Thoracoscopic | PVI + box | Clip |
| De Maat G^6^ | Thoracoscopic | PVI + box | No LAA management |
| Doty J^7^ | Thoracoscopic | PVI + box | Stapler |
| Driessen A^8^ | Thoracoscopic | PVI + box + epicardial lesions | Unspecified |
| Dunnington G^9^ | Hybrid | PVI + box + epicardial lesions + endocardial validation | Clip |
| Edgerton J^10^ | Thoracoscopic | PVI + box + epicardial lesions | Stapler |
| Gasbarri T^11^ | Hybrid | PVI + box + endocardial lesions | Clip |
| Geuzebroek G^12^ | Thoracoscopic | PVI + box + epicardial lesions | Stapler |
| Guo H^13^ | Hybrid | PVI + box + endocardial validation | Stapler |
| Haldar S^14^ | Thoracoscopic | PVI + box | Stapler + clip |
| Harlaar N^15^ (Leiden) | Thoracoscopic | PVI + box | Stapler + clip |
| Harlaar N^15^ (Eindhoven) | Thoracoscopic | PVI + box | Stapler + clip |
| Janusauskas V^16^ | Thoracoscopic | PVI + box + epicardial lesions | Suture |
| Ji Y^17^ | Thoracoscopic | PVI + box | Stapler |
| Khoynezhad A^18^ | Thoracoscopic | PVI + box + epicardial lesions | *NR* |
| Kim J^19^ | Thoracoscopic | PVI + box + epicardial lesions | Stapler |
| Lapenna E^20^ | Hybrid | PVI + box + endocardial lesions | No LAA management |
| Maesen B^21^ | Hybrid | PVI + box + endocardial lesions | Stapler + clip |
| Mahapatra S^22^ | Hybrid | PVI + box + epicardial lesions + endocardial lesions | Stapler |
| Mei J^23^ | Thoracoscopic | PVI + box + epicardial lesions | Stapler |
| Montero J^24^ | Thoracoscopic | PVI + box | Clip |
| Nasso G^25^ | Thoracoscopic | PVI + box + epicardial lesions | NR |
| Neefs J^26^ | Thoracoscopic | PVI + box + epicardial lesions | Stapler |
| Pannone L^27^ | Hybrid | PVI + box + endocardial lesions | Clip |
| Pearman C^28^ | Thoracoscopic | PVI + box | Clip |
| Pesl M^29^ | Hybrid | PVI +box + endocardial validation | *NR* |
| Pick A^30^ | Hybrid | PVI + box + endocardial validation | Clip |
| Pojar M^31^ | Thoracoscopic | PVI + box | Clip |
| Pokushalov E^32^ | Thoracoscopic | PVI + box | *NR* |
| Pong T^33^ | Hybrid | PVI + box + epicardial lesions + endocardial lesions | Clip |
| Probst J^34^ | Thoracoscopic | PVI + box + epicardial lesions | Stapler |
| Pruitt J^35^ | Hybrid | PVI + box + epicardial lesions + endocardial validation | Stapler |
| Richardson T^36^ | Hybrid | PVI + box + epicardial lesions + endocardial lesions | Clip |
| Rodrigues C^37^ | Thoracoscopic | PVI + box | *NR* |
| Rollin A^38^ | Hybrid | PVI + box + endocardial lesions | *NR* |
| Saini A^39^ | Thoracoscopic | PVI + box + epicardial lesions | Stapler + clip |
| Santini M^40^ | Thoracoscopic | PVI + box | Suture |
| Schenk S^41^ | Thoracoscopic | PVI + box | *NR* |
| Sirak J^42^ | Thoracoscopic | PVI + box + epicardial lesions | Clip |
| Suwalski P^43^ | Hybrid | PVI + box + endocardial validation | *NR* |
| Tan C^44^ | Hybrid | PVI + box + epicardial lesions + endocardial validation | Clip |
| Troubil M^45^ | Thoracoscopic | PVI + box | No LAA management |
| Van Der Heijden C^46^ | Hybrid | PVI + box + endocardial lesions | Stapler + clip |
| Wang J^47^ | Thoracoscopic | PVI + box | Stapler |
| Witkowska A^48^ | Hybrid | PVI + box + epicardial lesions + endocardial validation | Stapler |
| Yan T^49^ | Thoracoscopic | PVI + box + epicardial lesions | Stapler |
| Yu C^50^ | Thoracoscopic | PVI + box | Stapler |
| Zheng S^51^ | Thoracoscopic | PVI + box | Stapler |
| Zotov A^52^ | Thoracoscopic | PVI + box | Stapler |

| **Supplementary Table 5. Rationale for covariate selection in the long-term Cox-regression models for freedom from atrial tachyarrhythmias (ATA).** | |
| --- | --- |
| **Definitive model** *(Covariates)* | ***Rationale for covariate inclusion*** |
| Mean age (years) | Significantly different between patients undergoing thoracoscopic vs. hybrid thoracoscopic ablation. Older age seems to be an important predictor of ATA recurrences both after catheter and surgical ablation.^53, 54^ |
| Male sex (%) | Significantly different between patients undergoing thoracoscopic vs. hybrid thoracoscopic ablation. Literature reports show significant differences between men and women in outcomes after AF-ablation.^55^ Also, there are differences in the AF-substrate between male and female patients undergoing catheter ablations.^56^ |
| Paroxysmal AF history (%) | Significantly different between patients undergoing thoracoscopic vs. hybrid thoracoscopic ablation. Also, patients with persistent AF hold a more advanced arrhythmogenic substrate as compared to patients with paroxysmal AF.^57^ |
| AF duration (days, mean) | Significantly different between patients undergoing thoracoscopic vs. hybrid thoracoscopic ablation. Longer duration of AF-history also implies a longer exposure to the arrhythmogenic effects of AF on the progression of atrial remodeling.^57^ |
| Hybrid AF ablation (%) | Variable of interest. |
| Frailty term (study) | Correction for frailty (study variable) enables adjustment for study level differences which are not addressed by the variables included in the model. |
| *Abbreviations: ATA: atrial tachyarrhythmias, PAF: paroxysmal atrial fibrillation, CI: confidence interval, HR: hazard ratio, LVEF: left ventricular ejection fraction.* | |

| **Supplementary Table 6. Long-term freedom from ATA estimated from adjusted Cox proportional hazard model** | | | | | |
| --- | --- | --- | --- | --- | --- |
| **Set time points**  **during the FU** | **Overall**  **(N = 2 038)** | **Thoracoscopic**  **(N = 1 189)** | **Hybrid**  **(N = 849)** | **Adjusted Hazard**  **Ratio (95% CI)^a^** | **P-value** |
| 1-year freedom from ATA, in % | 76.6 | 71.6 | 82.0 | HR=0.43 (0.29-0.64) | **<0.001** |
| 3-year freedom from ATA, in % | 61.6 | 55.1 | 69.9 | HR=0.57 (0.42-0.79) | **<0.001** |
| 5-year freedom from ATA, in % | 53.6 | 46.8 | 63.6 | HR=0.59 (0.43-0.83) | **<0.001** |
| Data on freedom from ATA was obtained from Cox proportional hazard models with adjustment for age, sex, paroxysmal AF-history, and AF-duration.  *Abbreviations:* ATA: atrial tachyarrhythmia, CI: confidence interval, FU: follow-up, POAF: postoperative atrial fibrillation, SR: sinus rhythm  ^a^Reference category is thoracoscopic AF-ablation. | | | | | |

| **Supplementary Table 7. Adjusted Cox proportional hazard frailty model for ATA-recurrences** | | | | | |
| --- | --- | --- | --- | --- | --- |
| **Variable** | **Beta** | **P-value** | **Hazard ratio** | **95% CI for HR** | |
|  |  |  |  | **Lower** | **Upper** |
| Age, per 1 year | 0.08 | **0.001** | 1.08 | 1.03 | 1.13 |
| Males, per 1% increase | 0.02 | **<0.001** | 1.02 | 1.01 | 1.04 |
| Paroxysmal AF-history, per 1% increase | 0.01 | 0.137 | 1.00 | 0.99 | 1.01 |
| AF-duration, per 1 month increase | 0.01 | 0.723 | 1.00 | 0.99 | 1.00 |
| One stage hybrid AF-ablation | -0.48 | **0.005** | 0.62 | 0.44 | 0.87 |
| Two stage hybrid AF-ablation | -0.62 | **0.004** | 0.52 | 0.33 | 0.81 |
| Frailty term (study) |  | **0.031** |  |  |  |
| *Abbreviations:* AF: atrial fibrillation, CI: confidence interval, HR: hazard ratio | | | | | |

| **Supplementary Table 8.** Publication bias assessment | | | |
| --- | --- | --- | --- |
| **Parameters** | **Intercept for bias** | **SE for intercept** | **P-value** |
| *Patient characteristics* | | | |
| Age, in years | -4.784 | 2.161 | 0.051 |
| Male subjects, % | 0.700 | 0.222 | 0.683 |
| Body mass index, in kg/m^2^ | 3.234 | 2.886 | 0.284 |
| Paroxysmal atrial fibrillation, in % | -0.034 | 0.458 | 0.261 |
| Duration atrial fibrillation, in years | 4.002 | 1.484 | **0.017** |
| CHA2DS2-VASc score | 1.964 | 0.405 | 0.933 |
| Hypertension, in % | 0.165 | 0.415 | 0.845 |
| Diabetes mellitus, in % | -1.687 | 0.293 | 0.219 |
| Stroke history, in % | -1.343 | 0.179 | **0.002** |
| Prior pulmonary vein isolation, in % | -0.982 | 0.404 | 0.895 |
| LVEF, in % | 4.144 | 4.033 | 0.321 |
| Left atrial diameter, in mm | -1.942 | 3.576 | 0.596 |
| Procedural time, in minutes | 1.210 | 31.968 | **0.005** |
| Conversion to sternotomy, in % | -1.971 | 0.586 | **0.007** |
| *Short-term outcomes* | | | |
| Early mortality, in % | -3.820 | 0.332 | **0.009** |
| Early stroke, in % | -2.576 | 0.779 | 0.073 |
| Early reoperations, in % | -3.464 | 0.451 | 0.261 |
| Cardiac tamponade, in % | -1.904 | 1.074 | 0.175 |
| Any bleeding, in % | -2.526 | 0.183 | **0.006** |
| Pneumonia, in % | -3.728 | 0.534 | 0.586 |
| Pleural effusion, in % | -2.486 | 1.018 | 0.300 |
| Pericarditis, in % | -3.630 | 0.818 | 0.663 |
| Phrenic nerve palsy, in % | -4.397 | 0.762 | 0.902 |
| PM implantation, in % | -3.896 | 0.128 | **0.004** |
| *Abbreviations: LVEF: left ventricular ejection fraction, SE: standard error.* | | | |

**Supplementary Figure 1.**

**
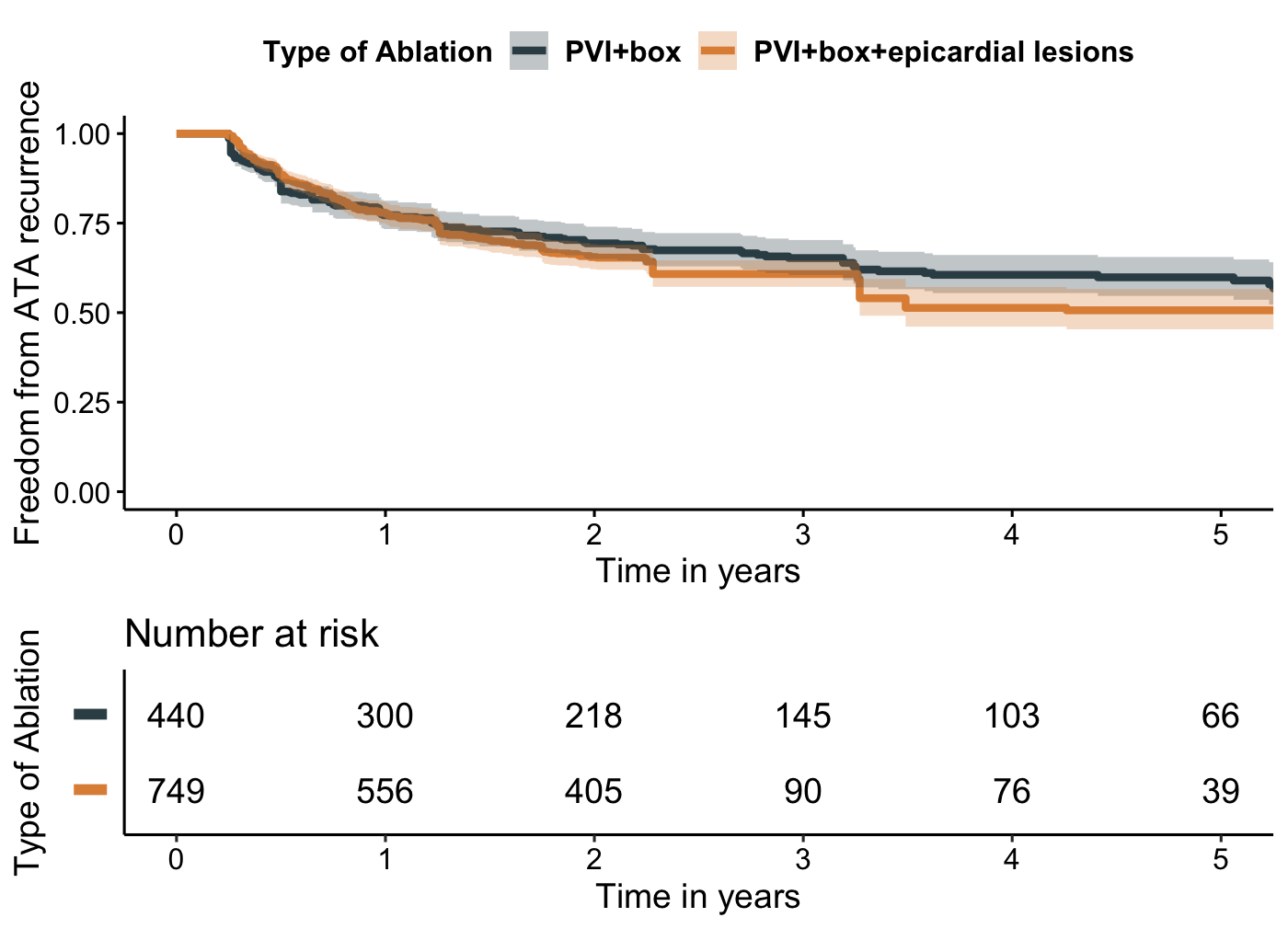
**

Effect of additional epicardial lesions on long-term freedom from ATA in patients undergoing isolated thoracoscopic ablation.

**Supplementary Figure 2.**

Kaplan Meier-curves for freedom from ATA for stand-alone thoracoscopic, one-stage and two-staged hybrid thoracoscopic-AF ablation. Adjusted for confounders including age, sex, paroxysmal AF history, duration of AF-history, and frailty term (study variable).

**Supplementary Material 2.** References.

[1] Al-Jazairi M, Rienstra M, Klinkenberg T, Mariani M, Van Gelder I, Blaauw Y. Hybrid atrial fibrillation ablation in patients with persistent atrial fibrillation or failed catheter ablation. *Netherlands Heart Journal* 2019; **27**: 142-151.

[2] An K, Yin H, Mei J, Zhu J, Tang M. Atrial endocardial expression of von Willebrand factor and thrombomodulin is associated with recurrence after minimally invasive surgical atrial fibrillation ablation. *Interact Cardiovasc Thorac Surg* 2018; **27**: 177-181.

[3] Arhire A, La Meir M, Blommaert D, Xhaet O, Deceuninck O, Collard E, et al. Hybrid ablation in refractory symptomatic persistent atrial fibrillation: one-step versus two-steps approach. *Acta Cardiologica: an international journal of cardiology* 2009; **64**: 697.

[4] R. B, M. B, C. S, M. K, J.-H. G-L, V. F. Bilateral video-assisted thoracoscopic maze procedure (mini-maze) for lone atrial fibrillation: Midterm results. *Innovations: Technology and Techniques in Cardiothoracic and Vascular Surgery* 2015; **10**: S55.

[5] C. Da, N. B, G. D, E. G, X. W, M. L, et al. Totally thoracoscopic surgical versus hybrid ablation of stand alone atrial fibrillation. *Archives of Cardiovascular Diseases Supplements* 2020; **12**: 112.

[6] De Maat GE, Van Gelder IC, Rienstra M, Quast A-FB, Tan ES, Wiesfeld AC, et al. Surgical vs. transcatheter pulmonary vein isolation as first invasive treatment in patients with atrial fibrillation: a matched group comparison. *Europace* 2014; **16**: 33-39.

[7] Doty JR, Clayson SE. Surgical treatment of isolated (lone) atrial fibrillation with Gemini-S Ablation and Left Atrial Appendage Excision (GALAXY procedure). *Innovations* 2012; **7**: 33-38.

[8] Driessen AH, Berger WR, Chan Pin Yin DR, Piersma FR, Neefs J, van den Berg NW, et al. Electrophysiologically guided thoracoscopic surgery for advanced atrial fibrillation: 5-year follow-up. *Journal of the American College of Cardiology* 2017; **69**: 1753-1754.

[9] Dunnington GH, Pierce CL, Eisenberg S, Bing LL, Chang-Sing P, Kaiser DW, et al. A heart-team hybrid approach for atrial fibrillation: a single-centre long-term clinical outcome cohort study. *European Journal of Cardio-Thoracic Surgery* 2021; **60**: 1343-1350.

[10] Edgerton JR, Jackman WM, Mack MJ. Minimally invasive pulmonary vein isolation and partial autonomic denervation for surgical treatment of atrial fibrillation. *Journal of Interventional Cardiac Electrophysiology* 2007; **20**: 89-93.

[11] T. G, G. A, D. G, E. K, S. B, V. B, et al. Minimally invasive hybrid sequential approach to persistent and long-standing persistent lone atrial fibrillation. *Innovations: Technology and Techniques in Cardiothoracic and Vascular Surgery* 2014; **9**: 194.

[12] Geuzebroek GS, Bentala M, Molhoek SG, Kelder JC, Schaap J, Van Putte BP. Totally thoracoscopic left atrial Maze: standardized, effective and safe. *Interactive cardiovascular and thoracic surgery* 2016; **22**: 259-264.

[13] Guo H, Qing H, Zhang Y, Liu J, Chen Z, Cao Z, et al. Stand-alone surgical ablation for atrial fibrillation: a novel bilateral double-port approach. *J Thorac Dis* 2019; **11**: 1989-1995.

[14] Haldar SK, Jones DG, Bahrami T, De Souza A, Panikker S, Butcher C, et al. Catheter ablation vs electrophysiologically guided thoracoscopic surgical ablation in long-standing persistent atrial fibrillation: the CASA-AF Study. *Heart Rhythm* 2017; **14**: 1596-1603.

[15] Harlaar N, Verberkmoes NJ, van der Voort PH, Trines SA, Verstraeten SE, Mertens BJ, et al. Clamping versus nonclamping thoracoscopic box ablation in long-standing persistent atrial fibrillation. *The Journal of thoracic and cardiovascular surgery* 2020; **160**: 399-405.

[16] Janusauskas V, Puodziukaite L, Maneikiene VV, Zuoziene G, Radauskaite G, Burneikaite G, et al. Long-term results of minimally invasive stand-alone bi-atrial surgical ablation with a bipolar ablation device for persistent and longstanding persistent AF: a single-center case series of 91 patients. *Journal of cardiothoracic surgery* 2016; **11**: 1-8.

[17] Ji Y, He L, Cheng Z, Shi J, Liu L, Guo Y. Five-year follow-up report: Box lesion radiofrequency ablation procedure for atrial fibrillation under video-assisted thoracoscope. *Clin Case Rep* 2021; **9**: e04837.

[18] A. K. Midterm outcomes of thoracoscopic ablation of atrial fibrillation using Dallas lesion set. *Innovations: Technology and Techniques in Cardiothoracic and Vascular Surgery* 2015; **10**: S55.

[19] Kim JY, Jeong DS, Kwon HJ, Park SJ, Park KM, Kim JS, et al. Effectiveness of the Early Staged Hybrid Approach for Treatment of Symptomatic Atrial Fibrillation: the Electrophysiology Study Could Be Deferred? *J Korean Med Sci* 2021; **36**: e276.

[20] Lapenna E, Cireddu M, Nisi T, Ruggeri S, Del Forno B, Monaco F, et al. Heart-team hybrid approach to persistent atrial fibrillation with dilated atria: the added value of continuous rhythm monitoring. *European Journal of Cardio-Thoracic Surgery* 2021; **60**: 222-230.

[21] Maesen B, Pison L, Vroomen M, Luermans JG, Vernooy K, Maessen JG, et al. Three-year follow-up of hybrid ablation for atrial fibrillation. *European Journal of Cardio-Thoracic Surgery* 2018; **53**: i26-i32.

[22] Mahapatra S, LaPar DJ, Kamath S, Payne J, Bilchick KC, Mangrum JM, et al. Initial experience of sequential surgical epicardial-catheter endocardial ablation for persistent and long-standing persistent atrial fibrillation with long-term follow-up. *The Annals of thoracic surgery* 2011; **91**: 1890-1898.

[23] Mei J, Ma N, Ding F, Chen Y, Jiang Z, Hu F, et al. Complete thoracoscopic ablation of the left atrium via the left chest for treatment of lone atrial fibrillation. *J Thorac Cardiovasc Surg* 2014; **147**: 242-246.

[24] L. M-C, D. P-C, B. T-M, M. C-A, F.J. C-C, L.C. M-C. Totally thoracoscopic left atrial maze procedure (TT-MAZE). *Cirugia Cardiovascular* 2020; **27**: 246-250.

[25] Nasso G, Lorusso R, Moscarelli M, De Martino G, Dell’Aquila AM, Motekallemi A, et al. Catheter, surgical, or hybrid procedure: what future for atrial fibrillation ablation? *Journal of Cardiothoracic Surgery* 2021; **16**: 186.

[26] Neefs J, Wesselink R, van den Berg NWE, de Jong J, Piersma FR, van Boven WP, et al. Thoracoscopic surgical atrial fibrillation ablation in patients with an extremely enlarged left atrium. *J Interv Card Electrophysiol* 2022; **64**: 469-478.

[27] Pannone L, Mouram S, Della Rocca DG, Sorgente A, Monaco C, Del Monte A, et al. Hybrid atrial fibrillation ablation: long-term outcomes from a single-centre 10-year experience. *Europace* 2023; **25**: euad114.

[28] Pearman CM, Redfern J, Williams EA, Snowdon RL, Modi P, Hall MC, et al. Early experience of thoracoscopic vs. catheter ablation for atrial fibrillation. *EP Europace* 2019; **21**: 738-745.

[29] Pešl M, Kulík T, Ostřížek T, Horváth V, Souček F, Melajová K, et al. Mid-term success rate of single stage hybrid ablation of persistent and long-term persistent atrial fibrillation. *Vnitrni Lekarstvi* 2022; **68**: 20-26.

[30] Pick AW, Kotschet E, Healy S, Adam D, Bittinger L. Hybrid Totally Thoracoscopic Maze and Catheter Ablation for Persistent Atrial Fibrillation: Initial Experience. *Heart Lung Circ* 2023; **32**: 1107-1114.

[31] Pojar M, Haman L, Parizek P, Myjavec A, Gofus J, Vojacek J. Midterm outcomes of mini-invasive surgical and hybrid ablation of atrial fibrillation. *Biomed Pap Med Fac Univ Palacky Olomouc Czech Repub* 2019; **163**: P233-240.

[32] E. P, A. R, D. E, A.V. B-P, D. L, S. B. Thoracoscopic approach in the treatment of patients with atrial fibrillation: 1-year follow-up through continuous subcutaneous monitoring. *Innovations: Technology and Techniques in Cardiothoracic and Vascular Surgery* 2014; **9**: 195.

[33] Pong T, Shah RL, Carlton C, Truong A, Fann B, Cyr K, et al. Hybrid Ablation for Atrial Fibrillation: Safety & Efficacy of Unilateral Epicardial Access*Seminars in Thoracic and Cardiovascular Surgery*. Elsevier 2023: 277-286.

[34] Probst J, Jidéus L, Blomström P, Zemgulis V, Wassberg E, Lönnerholm S, et al. Thoracoscopic epicardial left atrial ablation in symptomatic patients with atrial fibrillation. *EP Europace* 2016; **18**: 1538-1544.

[35] Pruitt JC, Lazzara RR, Ebra G. Minimally invasive surgical ablation of atrial fibrillation: the thoracoscopic box lesion approach. *Journal of Interventional Cardiac Electrophysiology* 2007; **20**: 83-87.

[36] Richardson TD, Shoemaker MB, Whalen SP, Hoff SJ, Ellis CR. Staged versus Simultaneous Thoracoscopic Hybrid Ablation for Persistent Atrial Fibrillation Does Not Affect Time to Recurrence of Atrial Arrhythmia. *J Cardiovasc Electrophysiol* 2016; **27**: 428-434.

[37] Rodrigues C, Silva M, Cerejo R, Portugal G, Cunha P, Rodrigues R, et al. SURGICAL ABLATION OF ATRIAL FIBRILLATION AND LEFT ATRIAL APPENDAGE OCCLUSION BY A TOTALLY VIDEOTHORACOSCOPIC APPROACH-NEW PARADIGM? *Portuguese Journal of Cardiac Thoracic and Vascular Surgery* 2021; **28**: 21-24.

[38] Rollin A, Mandel F, Grunenwald E, Mondoly P, Monteil B, Marcheix B, et al. Hybrid surgical ablation for persistent or long standing persistent atrial fibrillation: A French single centre experience*Annales de Cardiologie et d'Angéiologie*. Elsevier 2020: 86-92.

[39] Saini A, Hu YL, Kasirajan V, Han FT, Khan MZ, Wolfe L, et al. Long-term outcomes of minimally invasive surgical ablation for atrial fibrillation: a single-center experience. *Heart Rhythm* 2017; **14**: 1281-1288.

[40] Santini M, Loiaconi V, Tocco MP, Mele F, Pandozi C. Feasibility and efficacy of minimally invasive stand-alone surgical ablation of atrial fibrillation. A single-center experience. *Journal of Interventional Cardiac Electrophysiology* 2012; **34**: 79-87.

[41] Schenk S, Yokoyama S, Penicka I, Avots A, Fritzsche D. Stand-alone Atrial Fibrillation Surgery by Totally Thoracoscopic Ablation: We are Good, but why aren't we better? *The Thoracic and Cardiovascular Surgeon* 2015; **63**: OP19.

[42] Sirak JH, Schwartzman D. Interim results of the 5-box thoracoscopic maze procedure. *The Annals of thoracic surgery* 2012; **94**: 1880-1884.

[43] P. S, G. S, A. W, J. K, P. S, R. W, et al. Video-assisted pulmonary vein isolation for lone atrial fibrillation using irrigated bipolar radiofrequency system-mid-and long-term results. *Interactive Cardiovascular and Thoracic Surgery* 2011; **12**: S64-S65.

[44] Tan C, Zeng L-J, Shi H-F, Tian Y, Ma N, Liu H, et al. Intraprocedural arrhythmia termination as an end point for hybrid ablation in patients with long-standing persistent atrial fibrillation: a 2-year follow-up study. *Interactive CardioVascular and Thoracic Surgery* 2021; **33**: 43-50.

[45] Troubil M, Simek M, Juchelka J, Steriovsky A, Hajek R, Santavy P. Thoracoscopic epicardial ablation of atrial fibrillation: Safety, efficacy, single center experience. *Biomedical Papers* 2022.

[46] van der Heijden CA, Weberndörfer V, Luermans JG, Chaldoupi SM, van Kuijk SM, Vroomen M, et al. Hybrid ablation of atrial fibrillation: A unilateral left‐sided thoracoscopic approach. *Journal of Cardiac Surgery* 2022; **37**: 4630-4638.

[47] Wang J, Li Y, Shi J, Han J, Xu C, Ma C, et al. Minimally invasive surgical versus catheter ablation for the long-lasting persistent atrial fibrillation. *PLoS One* 2011; **6**: e22122.

[48] A. W, K. J, R.H. S, B. S, D. D, J.P. S, et al. Results of totally thoracoscopic ablation with autonomic ganglia ablation in patients with persistent and long-standing persistent atrial fibrillation. *Innovations: Technology and Techniques in Cardiothoracic and Vascular Surgery* 2015; **10**: S54-S55.

[49] Yan T, Zhu S, Chen N, Zhu M, Zhu K, Wei L, et al. An off-pump biatrial mini-maze procedure for long-standing persistent atrial fibrillation. *Eur J Cardiothorac Surg* 2022; **62**.

[50] Yu C, Li H, Zhang H, Zheng Z. Midterm results of stand-alone thoracoscopic epicardial ablation with box lesion for atrial fibrillation. *Interact Cardiovasc Thorac Surg* 2021; **33**: 354-361.

[51] Zheng S, Li Y, Han J, Zhang H, Zeng W, Xu C, et al. Long-term results of a minimally invasive surgical pulmonary vein isolation and ganglionic plexi ablation for atrial fibrillation. *PLoS One* 2013; **8**: e79755.

[52] Zotov A, Vachev S, Borisov D, Troitskiy A, Khabazov R. Thoracoscopic pulmonary vein and left atrial posterior wall isolation combined with left atrial appendage resection in patients with long-standing persistent atrial fibrillation. *Brazilian Journal of Cardiovascular Surgery* 2020; **35**: 22-27.

[53] Bahnson TD, Giczewska A, Mark DB, Russo AM, Monahan KH, Al-Khalidi HR, et al. Association Between Age and Outcomes of Catheter Ablation Versus Medical Therapy for Atrial Fibrillation: Results From the CABANA Trial. *Circulation* 2022; **145**: 796-804.

[54] MacGregor RM, Khiabani AJ, Bakir NH, Manghelli JL, Sinn LA, Carter DI, et al. Impact of age on atrial fibrillation recurrence following surgical ablation. *The Journal of thoracic and cardiovascular surgery* 2021; **162**: 1516-1528.e1511.

[55] Cheng X, Hu Q, Gao L, Liu J, Qin S, Zhang D. Sex-related differences in catheter ablation of atrial fibrillation: a systematic review and meta-analysis. *Europace : European pacing, arrhythmias, and cardiac electrophysiology : journal of the working groups on cardiac pacing, arrhythmias, and cardiac cellular electrophysiology of the European Society of Cardiology* 2019; **21**: 1509-1518.

[56] Wong GR, Nalliah CJ, Lee G, Voskoboinik A, Chieng D, Prabhu S, et al. Sex-Related Differences in Atrial Remodeling in Patients With Atrial Fibrillation: Relationship to Ablation Outcomes. *Circ Arrhythm Electrophysiol* 2022; **15**: e009925.

[57] Hindricks G, Potpara T, Dagres N, Arbelo E, Bax JJ, Blomström-Lundqvist C, et al. 2020 ESC Guidelines for the diagnosis and management of atrial fibrillation developed in collaboration with the European Association for Cardio-Thoracic Surgery (EACTS): The Task Force for the diagnosis and management of atrial fibrillation of the European Society of Cardiology (ESC) Developed with the special contribution of the European Heart Rhythm Association (EHRA) of the ESC. *Eur Heart J* 2021; **42**: 373-498.
