# Supplementary figures and images for: Short- and long-term outcomes in isolated vs. hybrid thoracoscopic ablation in patients with atrial fibrillation: a systematic review and reconstructed individual patient data meta-analysis
Source: Europace. 2024 Sep 10;26(10):euae232. doi: 10.1093/europace/euae232 (PMC11448334; doi:10.1093/europace/euae232)

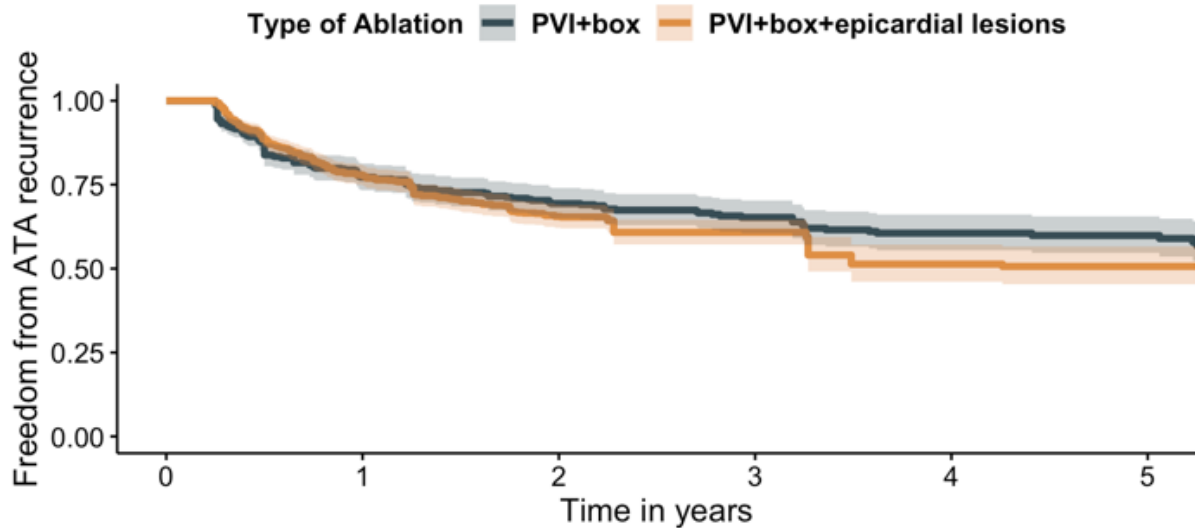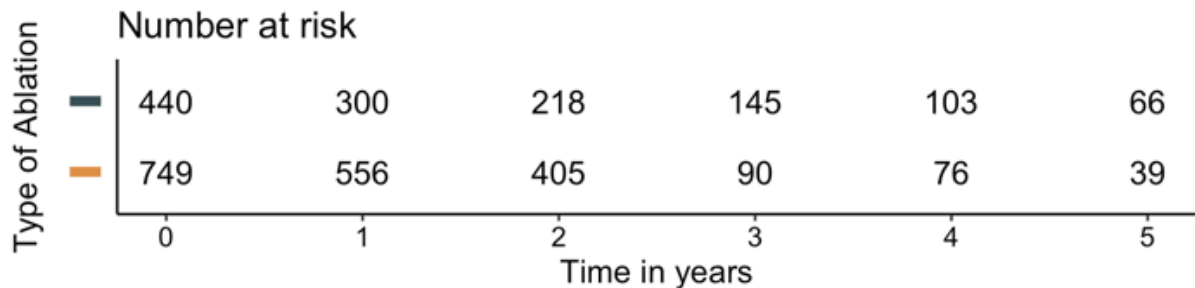

Supplement: euae232_Supplementary_Data [file euae232_supplementary_data.zip › Supplemental Figure 1.pdf]

Type of Ablation — Thoracoscopic — One-stage Hybrid — Two-stage Hybrid

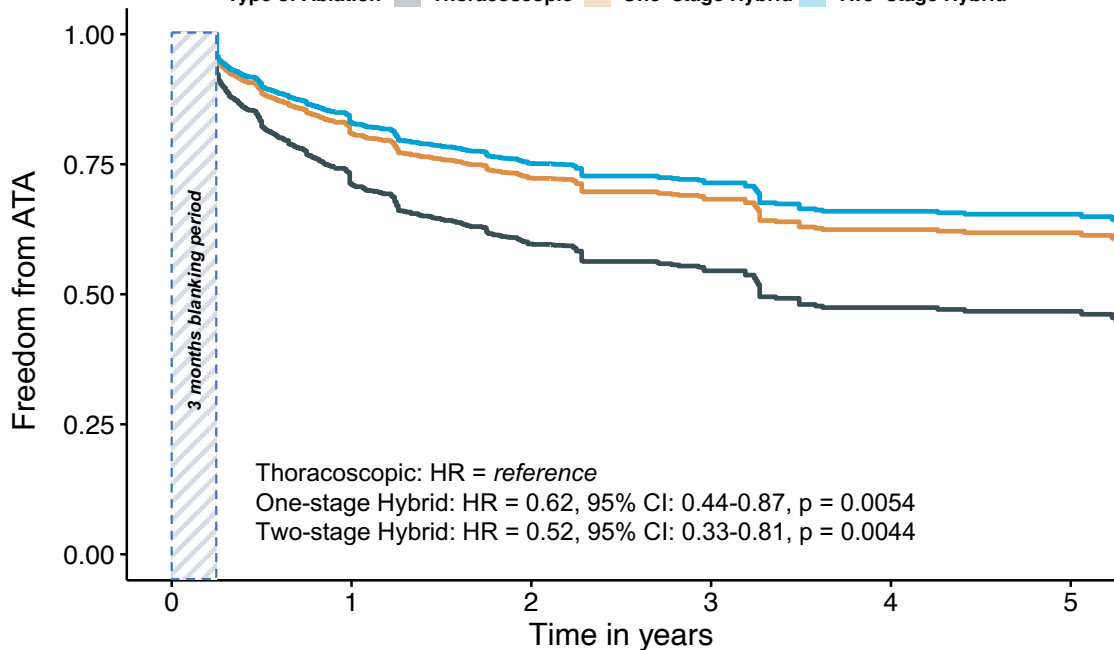

Supplement: euae232_Supplementary_Data [file euae232_supplementary_data.zip › Supplemental Figure 2.pdf]
